# DNA-Encoded Binding Affinity Shapes Robust CTCF Occupancy Across Cellular Contexts

**DOI:** 10.3390/biology15201843

**Published:** 2026-10-14

**Authors:** Zihang Yin, Yefan Wang, Zhiyu Zhang, Wuming Wang, Ya Guo

**Affiliations:** 1 Key Laboratory for the Genetics of Developmental and Neuropsychiatric Disorders (Ministry of Education), Joint International Research Laboratory of Metabolic and Developmental Sciences, School of Life Sciences and Biotechnology, Shanghai Jiao Tong University, Shanghai 200240, China; 2 Shanghai Pudong New Area People’s Hospital, Shanghai Jiao Tong University School of Medicine, Shanghai 201299, China; 3 School of Biomedical Sciences, Faculty of Medicine, The Chinese University of Hong Kong, Hong Kong, China

**Keywords:** CTCF, DNA-encoded affinity, chromatin occupancy, genome organization, A/B compartments, TADs, chromatin loops, cell-type specificity

## Abstract

CCCTC-binding factor (CTCF) is an architectural protein that organizes 3D genome architecture by binding to tens of thousands of genomic loci in a typical human cell type. Although DNA-binding affinity contributes to CTCF occupancy in vivo, the principles distinguishing robust from context-dependent occupancy remain incompletely understood. Here, we integrated quantitative measurements of CTCF-DNA binding affinity with genome-wide occupancy profiles. Using synthetic tandem arrays of CTCF sites with defined affinity levels, we experimentally tested whether DNA-encoded affinity establishes a predictable hierarchy of CTCF occupancy in vivo. We identified two CTCF-DNA binding affinity thresholds defining three distinct CTCF occupancy regimes. High-affinity sites were more frequently and consistently occupied across diverse cellular contexts and chromatin environments, whereas intermediate- and low-affinity sites showed greater context dependence. Synthetic arrays further revealed a reproducible occupancy hierarchy that was maintained across different motif arrangements. High-affinity sites were preferentially associated with strong chromatin loops and TAD boundaries, and flanking CTCF motifs that contribute to binding affinity were enriched at strong loop anchors. Together, these findings establish a quantitative framework in which CTCF-DNA binding affinity links sequence-encoded binding properties to the robustness and context dependence of CTCF occupancy and to features of CTCF-mediated 3D genome organization.

## Simple Summary

The human genome contains genetic instructions that must be precisely organized for cells to function properly. A central challenge in 3D genome biology is understanding why some binding sites for the architectural protein CTCF remain stable across cellular contexts, whereas others are more sensitive to changes. In this study, we found that DNA sequences encode quantitative information that predicts the robustness of CTCF occupancy. We found a nonlinear relationship between DNA-binding affinity and CTCF occupancy across diverse cellular contexts, with high-affinity sites exhibiting more robust occupancy. These findings provide new insights into how DNA sequence information contributes to the stability and flexibility of 3D genome organization and may facilitate the interpretation of disease-associated genetic variants and future efforts to design DNA elements with tunable genome-organizing properties.

## 1. Introduction

The three-dimensional (3D) organization of the human genome is essential for coordinating gene regulation, maintaining cell identity, and ensuring proper cellular function [1,2,3,4,5]. A central mechanism underlying 3D genome organization is cohesin-driven loop extrusion, in which the cohesin complex uses ATP hydrolysis to actively extrude DNA and generate long-range chromatin interactions, including enhancer–promoter communications [6,7,8,9,10,11]. CTCF, a sequence-specific DNA-binding architectural protein, cooperates with cohesin to shape these interactions [12]. CTCF-binding sites can act as directional barriers to cohesin-based loop extrusion, such that the presence, absence, and orientation of CTCF occupancy contribute to determining where loop extrusion is halted [13,14,15,16]. Through this mechanism, CTCF contributes to the formation of specific chromatin loops and the establishment of topologically associating domains (TADs) and their boundaries [16,17,18]. Thus, CTCF occupancy represents a critical regulatory layer through which DNA sequence information is translated into specific 3D genome structures.

The importance of CTCF-mediated genome organization is supported by its essential roles in development and cellular function. Genetic perturbation of CTCF disrupts early embryonic development and affects tissue-specific processes, including cardiac and limb development [19,20,21]. CTCF also contributes to transcriptional insulation, maintenance of genomic imprinting, and promoter choice within clustered protocadherin genes [13,22,23,24,25]. Because chromatin architecture differs between cell types, cell-type-specific CTCF occupancy is thought to contribute to the establishment of cell-type-specific 3D genome organization [26,27]. However, although CTCF can specifically recognize more than 110,000 human genomic sites [28], these sites exhibit different occupancy patterns across cellular contexts. Some sites are consistently occupied across diverse tissues and cell types, whereas others show substantial context-dependent variation in occupancy [29,30]. The molecular principles distinguishing constitutive from context-dependent CTCF occupancy remain incompletely understood.

CTCF occupancy is shaped by both DNA-encoded sequence information and the local chromatin environment [31,32]. CTCF recognizes DNA through its zinc-finger array, and sequence variation within and surrounding the core CTCF motif can substantially influence its in vivo occupancy [27,33,34,35,36,37,38]. CTCF occupancy can also be affected by DNA methylation, chromatin remodeling complex imitation switch (ISWI), and other features of the local chromatin environment [29,39,40,41,42,43,44]. These observations raise an important question: does DNA-encoded binding affinity primarily determine the probability of CTCF occupancy, or does it additionally determine how robustly occupancy is maintained when the chromatin environment changes? Stronger DNA binding could simply increase occupancy probability along a continuum. Alternatively, affinity may have a broader functional role, with distinct affinity ranges defining sites that differ in their sensitivity to cellular context. Determining whether and how affinity influences the stability of in vivo CTCF occupancy is therefore important for understanding how DNA-encoded information contributes to stable 3D genome architecture while permitting cell-type-specific remodeling.

Quantitative measurements of protein-DNA interactions across the genome provide an opportunity to address this question. In our recent study, we quantitatively measured CTCF-DNA binding affinity for approximately 270,000 human genomic sequences in vitro and observed moderate correlations between DNA-binding affinity and in vivo CTCF occupancy across several human tissues and cell lines (Spearman’s *ρ* ranging from 0.43 to 0.62) [28]. These findings established a quantitative relationship between DNA-encoded binding affinity and CTCF occupancy, but raised a further question: does this relationship simply reflect a continuum of binding probability, or does DNA-encoded affinity define distinct regimes of occupancy robustness? Furthermore, it remains unclear how affinity-dependent occupancy responds to changes in the chromatin environment, particularly across cellular contexts with distinct chromatin states.

Here, we first identified two affinity thresholds that delineate three CTCF occupancy regimes, with high-affinity sites showing robust occupancy across 61 diverse tissue and cellular contexts and intermediate- and low-affinity sites exhibiting progressively greater context dependence. Chromatin compartment switching was associated with greater changes in occupancy at lower-affinity sites, while synthetic tandem arrays of CTCF sites showed that high-affinity sites consistently exhibited higher occupancy across different motif arrangements. High-affinity sites were preferentially involved in strong chromatin loops and were enriched at TAD boundaries, whereas flanking CTCF motifs that contribute to binding affinity also show a tendency to be enriched at strong loop anchors. Together, these findings establish DNA-encoded binding affinity as a quantitative framework for understanding the robustness and context dependence of CTCF occupancy and provide a link between sequence-encoded CTCF binding properties and CTCF-based 3D genome organization.

## 2. Materials and Methods

### 2.1. Affinity-Occupancy Threshold Analysis

CTCF-DNA binding affinity data were downloaded from the open repository Zenodo (https://doi.org/10.5281/zenodo.21410880) [28]. CTCF-DNA binding affinities were measured in vitro using the high-throughput MpEMSA-seq approach. Mapped reads were first counted for each CTCF site and then normalized to one million total reads for the shifted (RPM^shift^) and input (RPM^input^) libraries, respectively. The relative affinity value of CTCF for each site was calculated as the ratio of RPM^shift^ to RPM^input^ (RPM^shift^/RPM^input^). ChIP-seq datasets from 61 ENCODE tissue and cellular contexts were obtained from the ENCODE project (Table S1) [29]. ChIP-seq datasets for 25 non-redundant cell types are listed in Table S2.

The relationship between DNA-encoded binding affinity and in vivo occupancy frequency was modeled using a self-starting logistic (SSlogis) function. CTCF occupancy was determined based on the ENCODE CTCF ChIP-seq peak annotations, with CTCF sites overlapping an identified peak classified as occupied. CTCF sites were ranked according to their measured CTCF-DNA binding affinity and divided into subsets with comparable numbers of sites. For each subset, the median affinity and corresponding median occupancy frequency were calculated and used as input for nonlinear regression. The SSlogis model was fitted in R (v3.6.1), and the first-, second-, and third-order derivatives of the fitted function were calculated to identify its transition points. The upper and lower transition points were used to define the affinity boundaries of the high- and low-affinity occupancy regimes, respectively. The same analysis was independently performed using 25 non-redundant cell types to evaluate the robustness of the identified affinity thresholds. To further assess the robustness and uncertainty of the identified thresholds, bootstrap and sensitivity analyses were performed. For bootstrap analysis, the retained sequences were resampled with replacement within each of the original affinity-ranked subsets while preserving the sample size of each subset. The group-level median occupancy frequencies were recalculated and the same nonlinear model was refitted for 1000 bootstrap iterations. The 2.5th and 97.5th percentiles of the resulting transition-point estimates were used to define the 95% bootstrap confidence intervals. Sensitivity analyses were performed by varying the number of affinity-ranked subsets and by using alternative nonlinear fitting strategies, including a free-floor model and ranking based on affinity values. Genomic coordinates used in this analysis were based on the GRCh37/hg19 reference genome assembly.

### 2.2. A/B Compartment and Subcompartment Analysis

A/B compartment annotations were inferred using the eigenvector function in Juicer from in situ Hi-C datasets [14,45]. Subcompartment annotations were obtained from previously published datasets [46]. CTCF ChIP-seq peaks were obtained from the ENCODE project [29]. For each CTCF site, the genomic location of its midpoint was used to assign the site to the corresponding A/B compartment or subcompartment region. CTCF occupancy was determined based on the ENCODE CTCF ChIP-seq peak annotations, with CTCF sites overlapping an identified peak classified as occupied. Genomic coordinates used in this analysis were based on the GRCh37/hg19 reference genome assembly.

### 2.3. Chromatin Loop and TAD Boundary Analysis

CTCF ChIA-PET datasets were downloaded from the UCSC database: https://hgdownload.soe.ucsc.edu/goldenPath/hg19/database/ (accessed on 21 February 2025). For each CTCF site, the midpoint of the site was used to determine whether it overlapped a loop anchor region. Genomic coordinates used in this analysis were based on the GRCh37/hg19 reference genome assembly.

TAD boundary annotations were obtained from previously published datasets [47]. To ensure consistency with the genomic coordinates of the TAD boundary annotations, the corresponding genomic coordinates of the CTCF sites were converted from hg19 to hg18. For each CTCF site, the midpoint of the site was used to determine whether it overlapped a TAD boundary region.

### 2.4. Cell Culture

HEK293T cells were cultured in DMEM (Thermo Fisher Scientific Inc., Waltham, MA, USA; 11995) containing 10% (*v*/*v*) FBS (Royacel Biotechnology Co., Ltd., Lanzhou, China; RYS-KF22) and 1% penicillin-streptomycin (Thermo Fisher Scientific Inc.; 1514). Cells were maintained at 37 °C with 5% CO_2_.

### 2.5. Molecular Cloning

A 1971 bp double-stranded DNA fragment containing three high-affinity CTCF sites in a tandem array was synthesized (Sangon Biotech Co., Shanghai, China) and cloned into the pLVX-Puro plasmid (Takara Bio USA, Inc., Mountain View, CA, USA; 632183), in which the IRES-Puro had been replaced by a Puro-T2A–ZsGreen1 fusion. Different CTCF recognition sequences were cloned into the vector by overlapping PCR using primers listed in Table S3. The presence of different CTCF recognition sequences was confirmed by Sanger sequencing.

### 2.6. Lentivirus Production and Transduction

The pLVX lentiviral vector DNA, together with the psPAX2 packaging and pMD2.G envelope plasmid DNA, was transfected into HEK293T cells (around 80% confluent) using a Calcium Phosphate Cell Transfection Kit (Beyotime Biotechnology Co., Ltd., Shanghai, China; C0508) to produce lentiviral particles. The harvested medium containing lentiviral particles was filtered through a 0.45 μm filter and frozen at −80 °C. For transduction, lentiviral particles were added to HEK293T cells in medium containing 8 μg/mL polybrene (Merck KGaA, Darmstadt, Germany; TR1003). Transduced cells were selected with 2 μg/mL puromycin (MedChemExpress, Shanghai, China; HY-B1743A) 24 h after infection. Uninfected cells were used as a negative control, and puromycin selection was discontinued once all uninfected control cells had died. The surviving transduced cells were maintained as pooled cell populations and used directly for subsequent experiments without clonal isolation. The exact lentiviral integration copy number was not determined.

### 2.7. ChIP-qPCR

Ten million cells were fixed at room temperature for 10 min using 1% formaldehyde (Thermo Fisher Scientific Inc.; 28908). Cross-linking was terminated by adding glycine solution (Merck KGaA; G5417) to a final concentration of 0.125 M. The resulting cell pellet was suspended in 1 mL ice-cold RIPA buffer containing 10 mM Tris (pH 7.5), 0.15 M NaCl, 1% Triton X-100, 1 mM EDTA, 0.1% sodium deoxycholate, 0.1% SDS, and 1× Roche EDTA-free protease inhibitors, followed by gentle rotation at 4 °C for 15 min. The RIPA treatment was performed twice, after which the nuclei were collected and resuspended in sonication buffer consisting of 10 mM Tris (pH 7.5), 0.15 M NaCl, 1% Triton X-100, 1 mM EDTA, 0.1% sodium deoxycholate, 1% SDS, and 1× Roche EDTA-free protease inhibitors. Chromatin was fragmented using a Scientz 08-III sonicator (Scientz Biotechnology Co., Ltd., Ningbo, China) at 90% amplitude for 25 min, with 25 cycles of 30 s on and 30 s off. Following sonication, the soluble chromatin fraction was diluted tenfold with dilution buffer containing 10 mM Tris (pH 7.5), 0.15 M NaCl, 1% Triton X-100, 1 mM EDTA, 0.1% sodium deoxycholate, and 1× Roche EDTA-free protease inhibitors. For immunoprecipitation, the diluted chromatin was incubated with an anti-CTCF antibody (Abcam, Cambridge, UK; ab70303) overnight at 4 °C with gentle rotation. The antibody-associated DNA-protein complexes were subsequently captured using magnetic beads (Thermo Fisher Scientific Inc.; 10004D). The beads were sequentially washed once with 1 mL ice-cold RIPA buffer containing 0.15 M NaCl, RIPA buffer containing 0.4 M NaCl, RIPA buffer lacking NaCl, LiCl buffer (0.25 M LiCl, 0.5% NP-40, 0.5% sodium deoxycholate, and 1× Roche EDTA-free protease inhibitors), and 1× Tris-EDTA buffer. Following reversal of the cross-links, DNA was isolated by phenol-chloroform-isoamyl alcohol extraction. Purified DNA concentration was measured with the Qubit dsDNA HS Assay Kit (Thermo Fisher Scientific Inc.; Q32854) using a Qubit fluorometer (Thermo Fisher Scientific Inc.).

DNA obtained from ChIP was quantified by real-time polymerase chain reaction using the FastStart Universal SYBR Green Master reagent (Roche, Basel, Switzerland; 4913914001) on a qTOWER^3^G Touch Real-Time PCR machine (Analytik Jena, Thuringia, Germany). Quantitative PCR reactions were performed in triplicate. Relative amplicon levels were determined from a standard curve generated from serially diluted input (lysates with no antibody added) DNA, and fold enrichment was calculated relative to a control region. All ChIP-qPCR data were obtained from two independent biological replicates. Relative CTCF occupancy measured by ChIP-qPCR across two biological replicates is presented in Table S4. The sequences of primers used for ChIP-qPCR are listed in Table S3.

## 3. Results

### 3.1. Identification of Two Affinity Thresholds That Define Three CTCF Occupancy Regimes

**Figure 1:**
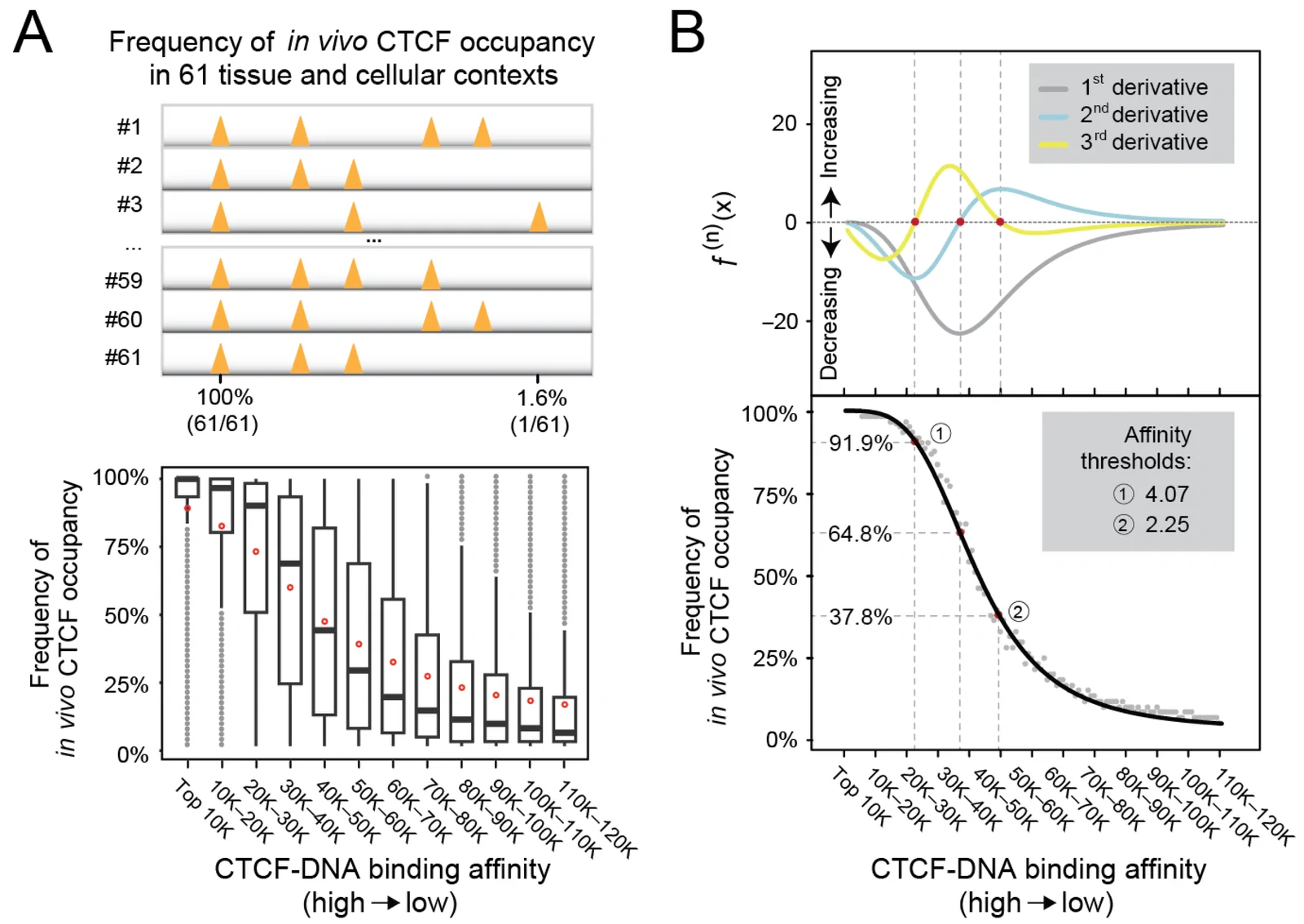
Identification of two affinity thresholds that define three CTCF occupancy regimes. (**A**) Schematic representation of in vivo CTCF occupancy frequency across 61 tissue and cellular contexts (top). CTCF recognition sequences were stratified into 12 intervals according to in vitro CTCF-DNA binding affinity (bottom). Boxplots show the relationship between DNA-encoded affinity and in vivo CTCF occupancy frequency. Boxes show the median (center line) and interquartile range (IQR; box), with the mean indicated by a red circle and whiskers extending to 1.5 × IQR. (**B**) Nonlinear regression analysis of the relationship between affinity and occupancy frequency (bottom) and the corresponding first-, second-, and third-order derivative curves (top). The fitted regression curve is shown in black and the original data points in gray. The two transition points, corresponding to affinity thresholds of 4.07 and 2.25, are indicated.

To investigate the relationship between CTCF-DNA binding affinity and in vivo CTCF occupancy, we first stratified 118,395 CTCF-recognition sequences into 12 affinity intervals (Figure 1A). The first 11 intervals each contained 10,000 sequences, whereas the remaining sequences were assigned to the 12th interval. These CTCF-recognition sequences exhibited significantly higher CTCF-DNA binding affinity than 393 randomly generated control sequences in a high-throughput in vitro binding assay, supporting their specific recognition by CTCF [28]. We next determined the frequency of in vivo CTCF occupancy for each CTCF site across 61 ENCODE tissue and cellular contexts using ChIP-seq datasets from the ENCODE project (Figure 1A; Table S1) [28]. Notably, 51.2% of sites in the highest-affinity interval (top 10,000 sequences) were occupied by CTCF in all 61 contexts, and 75% were occupied in at least 93% of these contexts. In contrast, more than 75% of sites in the lowest-affinity interval were occupied in fewer than 20% of the analyzed tissue and cellular contexts. CTCF occupancy frequency progressively decreased with decreasing affinity, indicating a quantitative relationship between DNA-encoded affinity and in vivo occupancy.

To identify affinity thresholds associated with distinct occupancy behaviors, we further ranked all CTCF sites by affinity and divided them into 120 subsets in descending order of affinity (Figure 1B). The median affinity and occupancy frequency of each subset were calculated, and the resulting relationship was fitted using a nonlinear regression model with a self-starting logistic (SSlogis) function. We then calculated the first-, second-, and third-order derivatives of the fitted curve to identify its transition points. The upper transition point occurred at an affinity value of 4.07, below which CTCF occupancy frequency began to decline sharply. Occupancy frequency continued to decrease until reaching the lower transition point at an affinity value of 2.25, with an intermediate transition point at 3.10. Importantly, the affinity thresholds of 4.07, 2.25, and 3.10 identified here are specific to the MpEMSA-seq affinity scale and are not intended as universal thresholds applicable across different affinity assays. These results suggest that the affinity-occupancy relationship could be divided into distinct functional regimes.

To assess the robustness of these affinity thresholds, we performed bootstrap and sensitivity analyses using alternative binning schemes and nonlinear fitting strategies. First, we performed a bootstrap analysis by resampling the retained sequences with replacement within each of the 120 original subsets, preserving the sample size of each subset. Across 1000 bootstrap iterations, the 95% confidence intervals for the two transition points were 4.02–4.12 and 2.22–2.28, respectively, encompassing the original thresholds of 4.07 and 2.25. We then varied the number of bins and divided the CTCF sites into 60 or 30 subsets. Using the same computational procedure, the two transition points were estimated at 4.077 and 2.256 for the 60-subset analysis and 4.076 and 2.256 for the 30-subset analysis, respectively (Figure S1A). These values were highly consistent with the thresholds obtained using 120 subsets. Next, we evaluated alternative nonlinear fitting strategies. Using a free-floor model, the two transition points were estimated at 3.94 and 2.27, respectively. When the sites were ranked using their original values rather than log-transformed values, the corresponding transition points were 4.00 and 1.76, respectively (Figure S1B).

Moreover, although the datasets used in our analysis were obtained from the ENCODE Project, differences in sequencing depth, experimental procedures, and other technical factors may influence CTCF peak identification. To further assess the robustness of the affinity thresholds while minimizing potential effects arising from differences in the number of identified CTCF sites, we restricted the analysis to the 33 tissue and cellular contexts in which 60,000–80,000 CTCF sites were identified. Nonlinear regression analysis was performed using the default fixed-floor model. The two transition points were estimated at 4.30 and 2.38, respectively, which were broadly consistent with the thresholds identified in the full dataset (Figure S1C).

Overall, the two major affinity thresholds remained broadly consistent across bootstrap resampling, alternative binning schemes, and nonlinear fitting strategies, supporting the robustness of the thresholds used in our analysis.

To further assess whether the observed relationship was influenced by repeated or related cellular contexts, we performed an independent analysis using 25 non-redundant cell types after removing repeated cell types and related tissue samples. Applying the same analysis yielded an upper transition point at an affinity value of 4.29, a lower transition point at 1.90, and an intermediate transition point at 2.97 (Figure S2). The preservation of the three-regime pattern despite substantial changes in the dataset composition supports the robustness of the affinity-dependent occupancy relationship.

Together, these results establish a quantitative relationship between CTCF-DNA binding affinity and in vivo occupancy frequency. High-affinity CTCF sites (affinity > 4.07) were most frequently occupied across diverse tissue and cellular contexts, whereas progressively lower-affinity sites showed increasingly restricted occupancy. The two affinity thresholds therefore define three CTCF occupancy regimes, providing a quantitative framework for distinguishing robust from context-dependent occupancy.

### 3.2. CTCF-DNA Binding Affinity Shapes Robust CTCF Occupancy Across Cell Types

**Figure 2:**
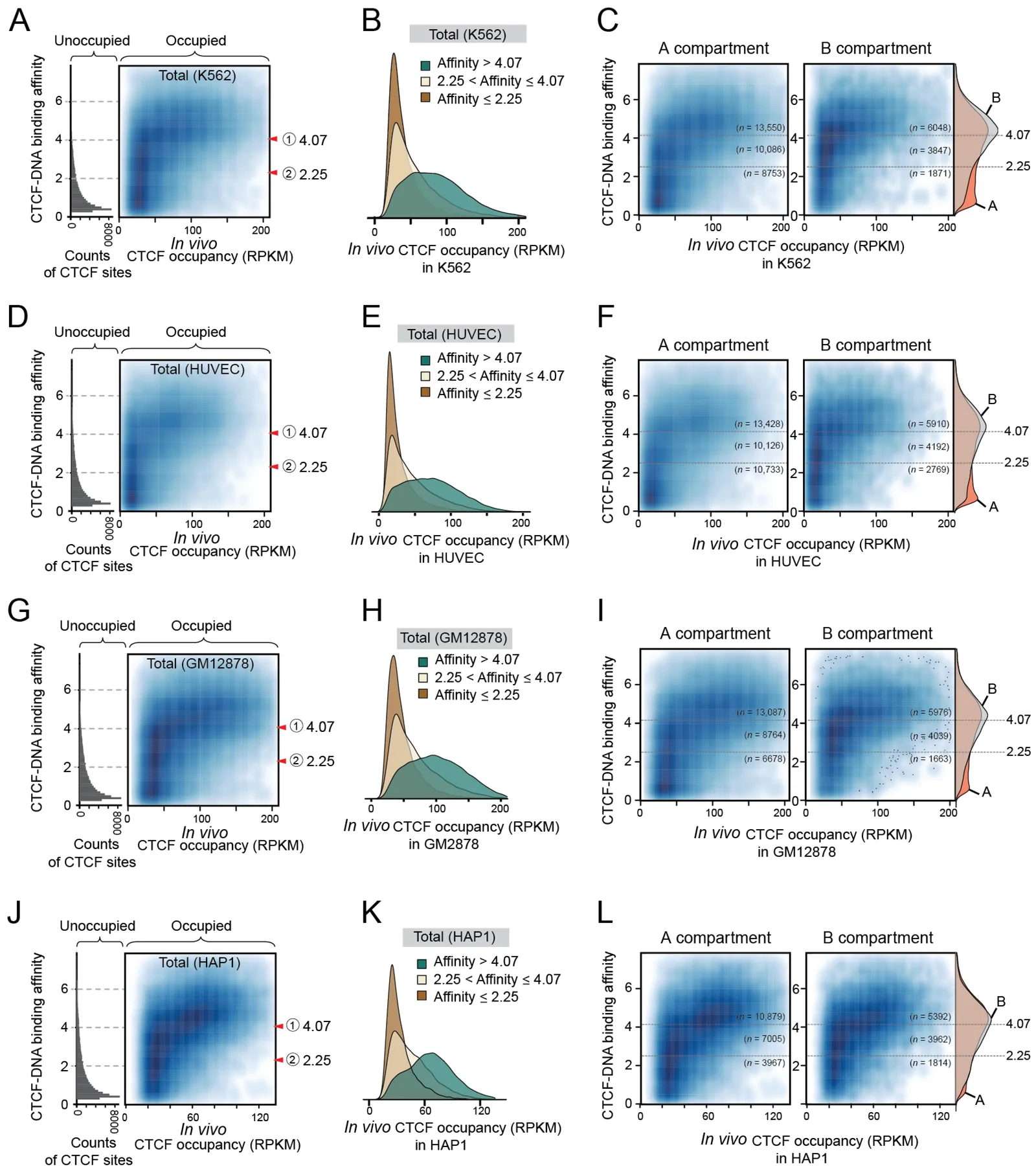
CTCF-DNA binding affinity shapes robust CTCF occupancy across K562, HUVEC, GM12878, and HAP1 cells. (**A**) The histogram in the left panel shows the distribution of CTCF-DNA binding affinity among unoccupied CTCF sites in human K562 cells. The smoothed scatter plot in the right panel shows the relationship between in vivo CTCF occupancy and in vitro CTCF-DNA binding affinity. In vivo CTCF occupancy is quantified by RPKM (reads per kilobase per million mapped reads), with the number of CTCF sites represented by color intensity. Two affinity thresholds (4.07 and 2.25) are indicated by red triangles. (**B**) Density plots show the overall distributions of CTCF sites across CTCF occupancy levels. CTCF sites are divided into three affinity groups: high-affinity sites (>4.07, dark green), intermediate-affinity sites (>2.25 and ≤4.07, yellow), and low-affinity sites (≤2.25, brown). (**C**) CTCF-occupied sites located in A-compartment (left panel) or B-compartment (middle panel) regions are shown separately. The right panel shows the normalized distributions of CTCF sites annotated to A (orange) or B (gray) compartments. The number of CTCF sites in each affinity group is indicated in parentheses. (**D**–**L**) Same as (**A**–**C**), showing the corresponding analyses in human HUVEC, GM12878, and HAP1 cells.

To investigate the relationship between CTCF-DNA binding affinity and in vivo occupancy within individual cell types, we generated two-dimensional density maps by plotting in vivo CTCF occupancy signals against the corresponding DNA-encoded CTCF-binding affinities at individual CTCF-occupied sites. In human K562 cells, we identified 44,155 CTCF-occupied sites, of which 44.4% were high-affinity sites, 31.5% were intermediate-affinity sites, and 24.1% were low-affinity sites (Figure 2A). In contrast, among 71,915 CTCF recognition sequences with significantly higher binding affinity than control random sequences but without detectable CTCF occupancy, only 3.7% were high-affinity sites, whereas 15.6% were intermediate-affinity and 80.7% were low-affinity sites. Notably, 88.1% of high-affinity CTCF sites were occupied in K562 cells, consistent with their high occupancy frequency observed across diverse cellular contexts. By comparison, 55.4% of intermediate-affinity sites and only 15.5% of low-affinity sites were occupied. Moreover, high-affinity CTCF sites not only exhibited a higher occupancy frequency but also showed stronger overall occupancy signals, whereas low-affinity sites generally displayed the weakest overall occupancy signals (Figure 2B).

Previous Hi-C studies have established that the genome can be broadly partitioned into A and B compartments according to patterns of three-dimensional chromatin interactions [14,48]. A compartments are generally associated with more open chromatin, higher levels of active chromatin marks, and transcriptionally active regions, whereas B compartments are generally associated with more compact chromatin and transcriptionally inactive or repressed regions. To examine the relationship between DNA-encoded CTCF-binding affinity, CTCF occupancy, and chromatin compartment state, we further stratified CTCF-occupied sites according to their localization within A or B compartments (Figure 2C). Low-affinity CTCF sites were more frequently occupied in A compartments (8753 sites) than in B compartments (1871 sites). Nevertheless, high-affinity sites consistently exhibited the strongest CTCF occupancy in both A and B compartments, although overall CTCF occupancy signals were higher in A compartments (Figure S3A).

A and B compartments can be further subdivided into eight subcompartments, designated A1, A2, A3, A4, B1, B2, B3, and B4 [46,49]. Across these subcompartments, active chromatin signals, such as H3K27ac, generally decrease from A1 toward B4, whereas the heterochromatin-associated mark H3K9me3 shows the opposite trend. This graded chromatin environment provides an opportunity to examine whether CTCF occupancy varies with DNA-encoded binding affinity across progressively less active chromatin states. We thus stratified CTCF-occupied sites according to their subcompartment annotations and quantified the number of CTCF-occupied sites within 100 kb genomic windows (Figure S4). In K562 cells, the number of occupied low-affinity CTCF sites progressively decreased from A1 to B4. In contrast, the numbers of occupied high- and intermediate-affinity sites remained relatively similar across A1-A3 and subsequently showed an overall decreasing trend toward the more repressive subcompartments. These results suggest that CTCF occupancy is associated with the local chromatin compartment state, with lower-affinity sites showing greater variation across the chromatin landscape. Nevertheless, CTCF-DNA binding affinity remains an important determinant of CTCF occupancy, even across different chromatin subcompartments.

We observed similar relationships between CTCF-DNA binding affinity and in vivo occupancy in HUVEC and GM12878 cells (Figure 2D–I and Figures S3B,C and S4), indicating that the association between sequence-encoded affinity and CTCF occupancy is not restricted to a single cellular context. To further assess whether genomic ploidy affected this relationship, we analyzed CTCF occupancy in the haploid human HAP1 cell line, excluding data from non-haploid chromosomes. The same overall relationship between CTCF-DNA binding affinity and CTCF occupancy was observed in HAP1 cells (Figure 2J–L and Figures S3D and S4).

Together, these results indicate that DNA-encoded binding affinity is consistently associated with in vivo CTCF occupancy and is an important feature underlying the robustness of CTCF occupancy across diverse cellular contexts.

### 3.3. Chromatin Compartment-State Differences Are Associated with Occupancy Changes at Lower-Affinity CTCF Sites

**Figure 3:**
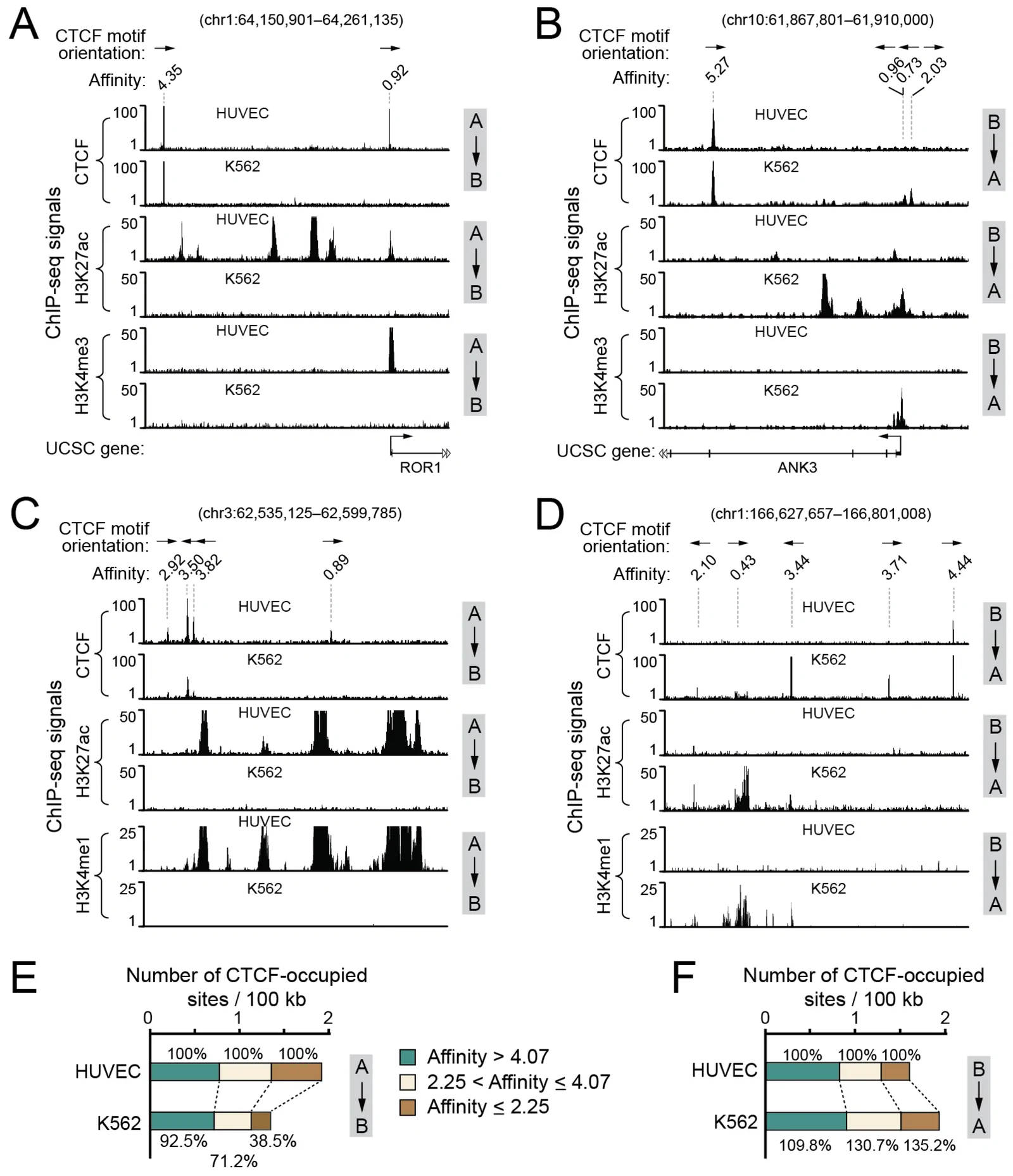
Chromatin compartment-state differences are associated with occupancy changes at lower-affinity CTCF sites. (**A**–**D**) Representative ChIP-seq profiles of four genomic regions exhibiting opposite compartment states between human HUVEC and K562 cells. Regions containing the *ROR1* (**A**) or *ANK3* (**B**) gene promoter show CTCF occupancy, H3K27ac, and H3K4me3 signals. Two enhancer regions on chr3 (**C**) and chr1 (**D**) show CTCF occupancy, H3K27ac, and H3K4me1 signals. (**E**,**F**) Bar plots showing the relative changes in the number of CTCF-occupied sites within genomic regions undergoing compartment switching. The horizontal arrows indicate the orientation of the CTCF motif corresponding to each CTCF site.

To further investigate how A/B compartment status influences CTCF occupancy, we analyzed changes in occupancy at CTCF sites located within genomic regions undergoing A/B compartment switching. For example, the promoter and surrounding region of the *ROR1* gene were annotated as an A compartment in HUVEC cells and contained several sites with H3K27ac signals indicative of open and active chromatin, while the *ROR1* promoter region was enriched for H3K4me3 (Figure 3A). In contrast, the same region was annotated as a B compartment in K562 cells, where H3K27ac signals were not readily detectable and H3K4me3 enrichment at the *ROR1* promoter region was undetectable. The *ROR1* promoter region contains a CTCF-binding site with an affinity score of 0.92, corresponding to a low-affinity site. This site was occupied by CTCF in HUVEC cells but not in K562 cells. Conversely, the promoter and surrounding region of the *ANK3* gene underwent the opposite compartment transition, from B in HUVEC cells to A in K562 cells (Figure 3B). Three CTCF sites located near the *ANK3* promoter had affinity scores of 0.96, 0.73, and 2.03, respectively, all corresponding to low-affinity sites. These three sites exhibited detectable CTCF occupancy only in K562 cells.

Consistent with these observations, CTCF occupancy also changed alongside A/B compartment switching at two representative enhancer regions marked by H3K27ac and H3K4me1. In a genomic region on chromosome 3 that underwent an A-to-B transition, four intermediate- or low-affinity CTCF sites showed markedly reduced CTCF occupancy (Figure 3C). In contrast, in a region on chromosome 1 that underwent a B-to-A transition, intermediate- and low-affinity sites showed significantly increased CTCF occupancy (Figure 3D).

Notably, high-affinity sites in these representative regions appeared relatively insensitive to A/B compartment switching. For example, a CTCF site upstream of the *ROR1* promoter had an affinity score of 4.35 and exhibited comparable CTCF occupancy in HUVEC and K562 cells, with strong CTCF enrichment detected in both cell types (Figure 3A). Moreover, we noted that some CTCF sites in these representative regions were directly located within promoter or enhancer regions and were associated with the occupancy of weaker CTCF sites. To systematically examine this relationship, we calculated the proportions of high-, intermediate-, and low-affinity CTCF sites located within active regulatory elements, defined by H3K27ac peaks, in K562 and GM12878 cells. In both cell types, the proportion of low-affinity CTCF sites located within active elements was at least 3.4-fold higher than that of high-affinity sites (Figure S3E,F). These observations suggested that high-affinity CTCF sites may be relatively resistant to changes in chromatin compartment state, whereas lower-affinity sites are more responsive to changes in their chromatin environment.

We next systematically quantified CTCF occupancy across all CTCF sites located within regions undergoing A/B compartment switching between HUVEC and K562 cells (Figure 3E,F). Occupancy was defined based on whether a site overlapped a detectable CTCF ChIP-seq peak. Following an A-to-B transition, the number of occupied high-affinity CTCF sites decreased only modestly, by 7.5%, whereas occupancy decreased by 28.8% and 61.5% at intermediate- and low-affinity sites, respectively (Figure 3E). Conversely, following a B-to-A transition, the number of occupied high-affinity sites increased by only 9.8%, compared with increases of 30.7% and 35.2% at intermediate- and low-affinity sites, respectively (Figure 3F). Thus, changes in chromatin compartment state were associated with substantially greater changes in CTCF occupancy at lower-affinity sites than at high-affinity sites.

**Figure 4:**
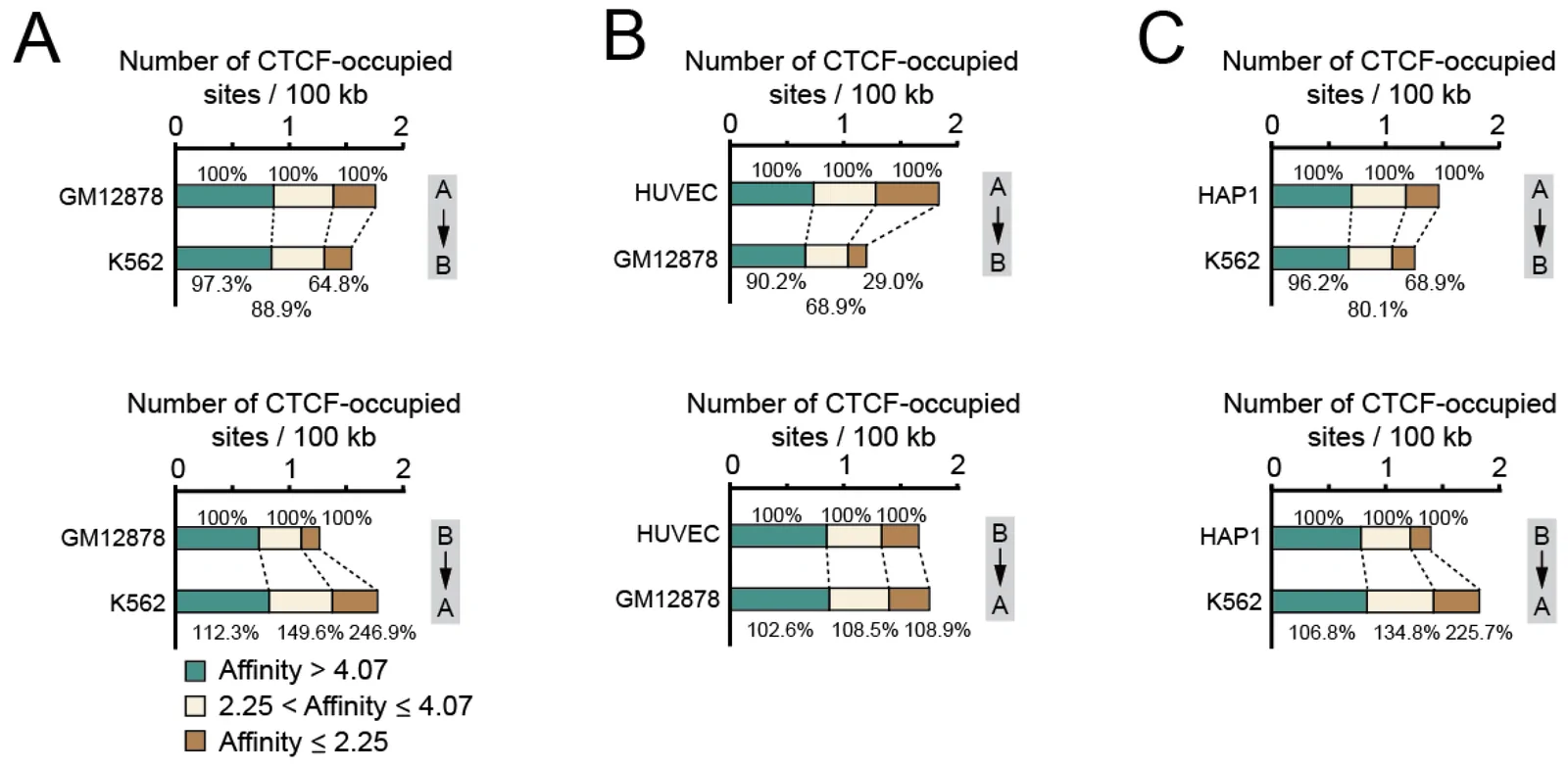
Changes in CTCF occupancy numbers associated with A/B compartment switching. Bar plots show the percentage changes in the number of CTCF-occupied sites within genomic regions undergoing compartment switching between GM12878 and K562 cells (**A**), HUVEC and GM12878 cells (**B**), and HAP1 and K562 cells (**C**). The top panels show changes associated with A-to-B compartment transitions, whereas the bottom panels show changes associated with B-to-A compartment transitions.

This affinity-dependent pattern was not restricted to the HUVEC-K562 comparison. Similar trends were observed in comparisons between GM12878 and K562 cells, HUVEC and GM12878 cells, and HAP1 and K562 cells, with lower-affinity CTCF sites showing greater changes in occupancy following A/B compartment switching than high-affinity sites (Figure 4).

Together, these results suggest that CTCF-DNA binding affinity contributes to differential sensitivity of CTCF occupancy to changes in chromatin compartment state, with high-affinity sites exhibiting greater occupancy robustness and lower-affinity sites showing greater context dependence.

### 3.4. The Tested High-Affinity Site Exhibits More Robust CTCF Occupancy than Lower-Affinity Sites

**Figure 5:**
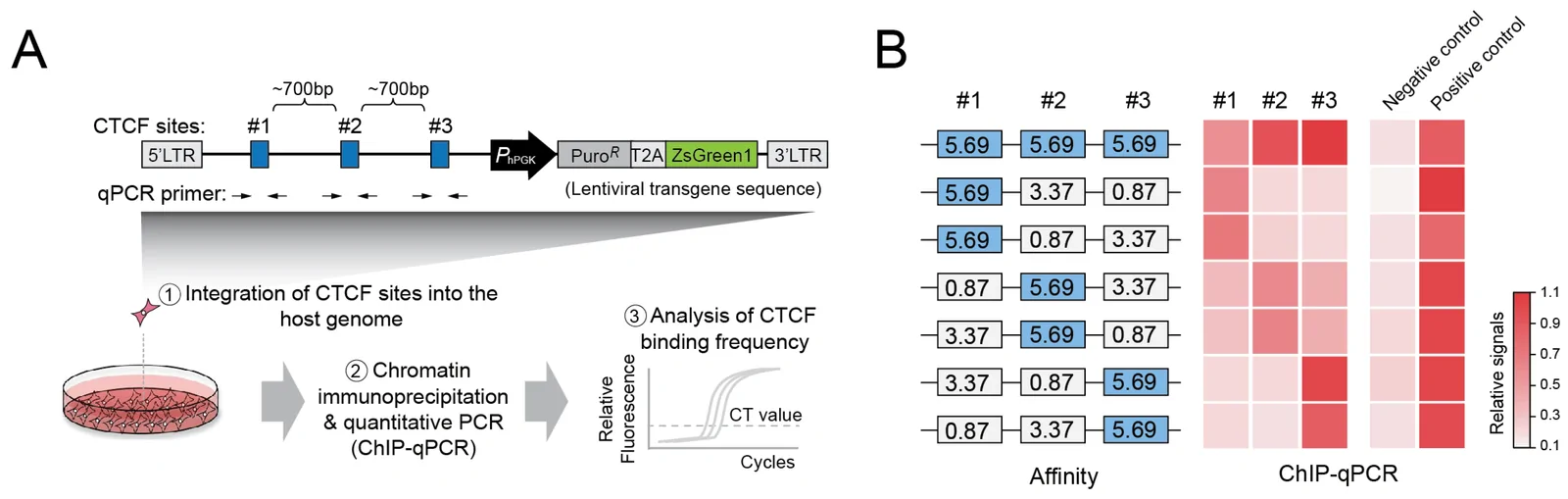
The tested high-affinity site exhibits more robust CTCF occupancy than lower-affinity sites. (**A**) Schematic illustrating the workflow for ChIP-qPCR to quantitatively measure relative CTCF occupancy. The horizontal arrows indicate the positions of the qPCR primer pairs. (**B**) Quantitative analysis of CTCF occupancy at three CTCF sites arranged in tandem and designated #1, #2, and #3, respectively. The affinity scores are shown in the left panel, with the high-affinity site (affinity score = 5.69) highlighted in blue. Heatmaps in the right panel show the mean relative CTCF occupancy measured by ChIP-qPCR across the three tandem sites and two control loci from two independent biological replicates (*n* = 2), with consistent trends observed between the two replicates. The red color indicates relative signal intensity, with darker shades representing stronger signals. The three sequences were tested in all six possible positional arrangements, and an H–H–H construct containing three identical copies of the high-affinity sequence was included as a control.

Next, we experimentally quantified CTCF binding to genomic sequences spanning a range of DNA-encoded binding affinities. We constructed a lentiviral reporter in which three CTCF-recognized sequences (#1, #2, and #3) were arranged in tandem, with approximately 700 bp of intervening sequence between adjacent sites to minimize potential interference between neighboring binding sites (Figure 5A and Figure S5). A hPGK promoter-driven Puro-T2A-ZsGreen1 expression cassette was placed downstream of the tandem CTCF sites to enable selection and identification of successfully transduced cells. The entire construct was introduced into the genome of HEK293T cells using a lentiviral system, and successfully transduced cells were selected with puromycin. CTCF occupancy at each of the three integrated sites was then quantitatively measured by ChIP-qPCR, and the relative occupancy strength was calculated for each site. In parallel, we examined CTCF occupancy at two fixed genomic loci as internal controls: one previously characterized CTCF-binding site served as a positive control, whereas a genomic locus known to lack CTCF binding served as a negative control.

To assess the contribution of DNA-encoded binding affinity to CTCF occupancy in vivo, we selected three sequences with experimentally measured CTCF-binding affinities of 5.69, 3.37, and 0.87, representing high-, intermediate-, and low-affinity sites, respectively (Figure 5B, left panel). All three sequences were devoid of CG dinucleotides to minimize potential effects of CpG methylation. To determine whether the relative position within the tandem array affected CTCF occupancy, the three sequences were systematically arranged in all six possible permutations across the three tandem sites: high–intermediate–low (H–M–L), high–low–intermediate (H–L–M), low–high–intermediate (L–H–M), intermediate–high–low (M–H–L), intermediate–low–high (M–L–H), and low–intermediate–high (L–M–H). In addition, the high-affinity sequence was placed at all three positions to generate an H–H–H construct as a positive control.

The negative-control genomic locus consistently exhibited low CTCF ChIP-qPCR signals across the different constructs, whereas the positive-control locus showed robust CTCF occupancy, demonstrating the reproducibility and dynamic range of the assay (Figure 5B, right panel). The H–H–H construct also exhibited high CTCF occupancy at all three integrated sites, indicating that all three positions within the tandem array were capable of supporting robust CTCF occupancy (Figure 5B, middle panel). Notably, across all six sequence arrangements, the high-affinity sequence consistently exhibited the strongest CTCF occupancy, regardless of its relative position within the tandem array. In contrast, the intermediate- and low-affinity sequences showed substantially weaker occupancy, with these lower-affinity sequences in some configurations displaying occupancy levels comparable to those observed at the genomic negative-control locus.

Together, these results provide experimental evidence that CTCF-DNA binding affinity is an important determinant of robust CTCF occupancy in vivo.

### 3.5. High-Affinity CTCF Sites Preferentially Participate in Chromatin Loops and Are Enriched at TAD Boundaries

**Figure 6:**
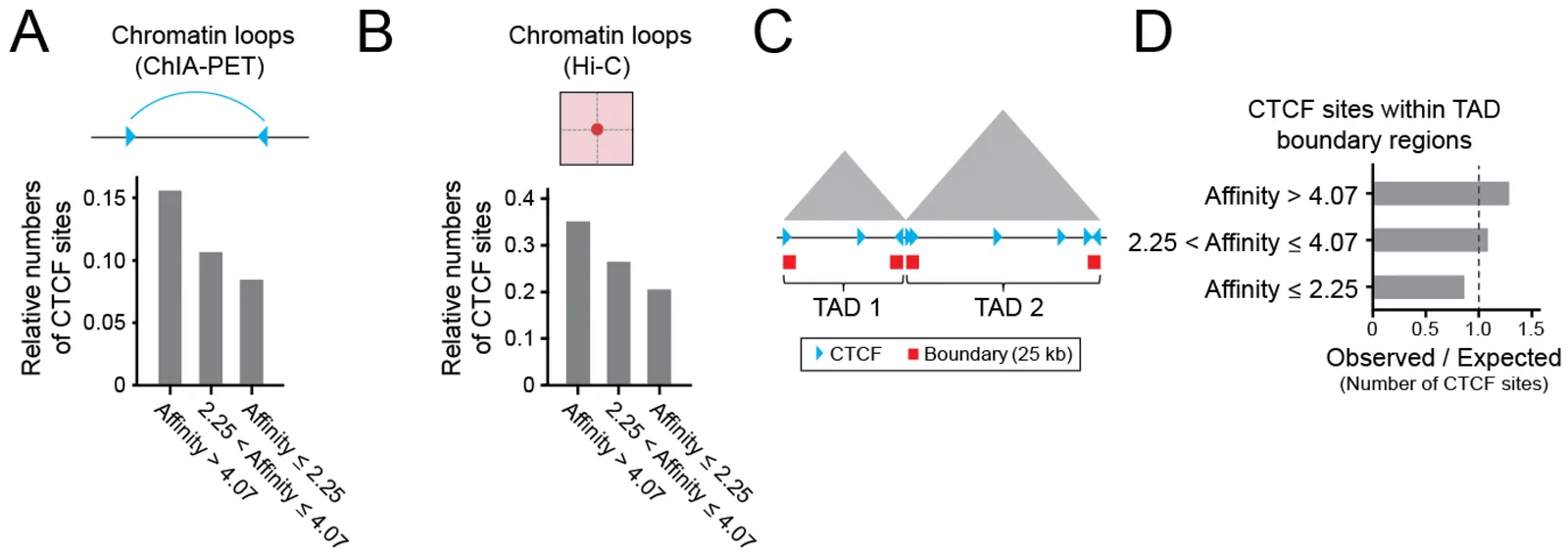
High-affinity CTCF sites preferentially participate in chromatin loops and are enriched at TAD boundaries. (**A**,**B**) High-affinity CTCF sites preferentially participate in strong chromatin loops in K562 cells, as identified by CTCF ChIA-PET (**A**) and Hi-C (**B**). (**C**) Schematic illustrating the distribution of CTCF sites within two topologically associating domains (TADs) and their boundary regions. (**D**) Observed-to-expected (O/E) ratios of high-, intermediate-, and low-affinity CTCF sites within TAD boundary regions in IMR90 cells [47].

Building on previous studies showing that CTCF occupancy and binding orientation contribute to chromatin interactions [14,15,16,50], together with our finding that high-affinity CTCF sites are more frequently occupied and exhibit stronger occupancy signals, we next asked whether high-affinity sites preferentially participate in stronger chromatin loops. We first analyzed CTCF ChIA-PET data from K562 cells [17,27], which are designed to capture CTCF-associated chromatin interactions and provide high-resolution information on interaction anchors. We defined strong chromatin loops as those with loop scores ≥ 500 and convergent CTCF motifs at the loop anchors (*n* = 3718). Within these strong loops, high-affinity CTCF sites were more frequently involved in chromatin interactions than intermediate- and low-affinity sites, with 47% and 85% higher frequencies, respectively (Figure 6A). We next examined strong chromatin loops identified by in situ Hi-C at 5 kb resolution. Defining strong loops as those within the top 20% of loops ranked by loop strength (*n* = 668), we again found that high-affinity CTCF sites were more frequently involved in chromatin interactions, with frequencies 33% and 70% higher than those of intermediate- and low-affinity sites, respectively (Figure 6B). Moreover, to minimize the potential influence of differences in CTCF occupancy, we further restricted the analysis to 276 strong ChIA-PET loops whose two anchor sites exhibited comparable relative CTCF ChIP-seq signals, ranging from 60 to 90. Within these loops, high-affinity CTCF sites were still more frequently involved in chromatin interactions than intermediate- and low-affinity sites, with 19% and 66% higher frequencies, respectively. Thus, across both CTCF ChIA-PET and Hi-C datasets, high-affinity CTCF sites are preferentially involved in strong chromatin loops.

Our previous work showed that CTCF occupancy and binding orientation are associated with TAD boundary formation [16]. Given that TAD structures are frequently conserved across different cell types [47], we reasoned that the more robust occupancy of high-affinity CTCF sites might also be associated with their preferential localization at TAD boundaries. To test this, we analyzed the observed and expected numbers of high-, intermediate-, and low-affinity occupied CTCF sites within TAD boundary regions, defined as 25 kb regions flanking each TAD boundary (Figure 6C). The expected number of sites for each affinity group was calculated based on its overall distribution among occupied CTCF sites, and enrichment was quantified as the observed-to-expected (O/E) ratio (Figure 6D). High-affinity CTCF sites were enriched by 28.5% at TAD boundaries (O/E = 1.285), whereas intermediate-affinity sites showed a more modest enrichment of 8.2% (O/E = 1.082). In contrast, low-affinity sites were depleted by 13.6% (O/E = 0.864). These results indicate that high-affinity CTCF sites are preferentially localized at TAD boundaries, further supporting an association between CTCF-DNA binding affinity and higher-order genome organization.

### 3.6. Flanking CTCF Motifs Are Associated with Robust CTCF Occupancy and Strong Chromatin Loops

**Figure 7:**
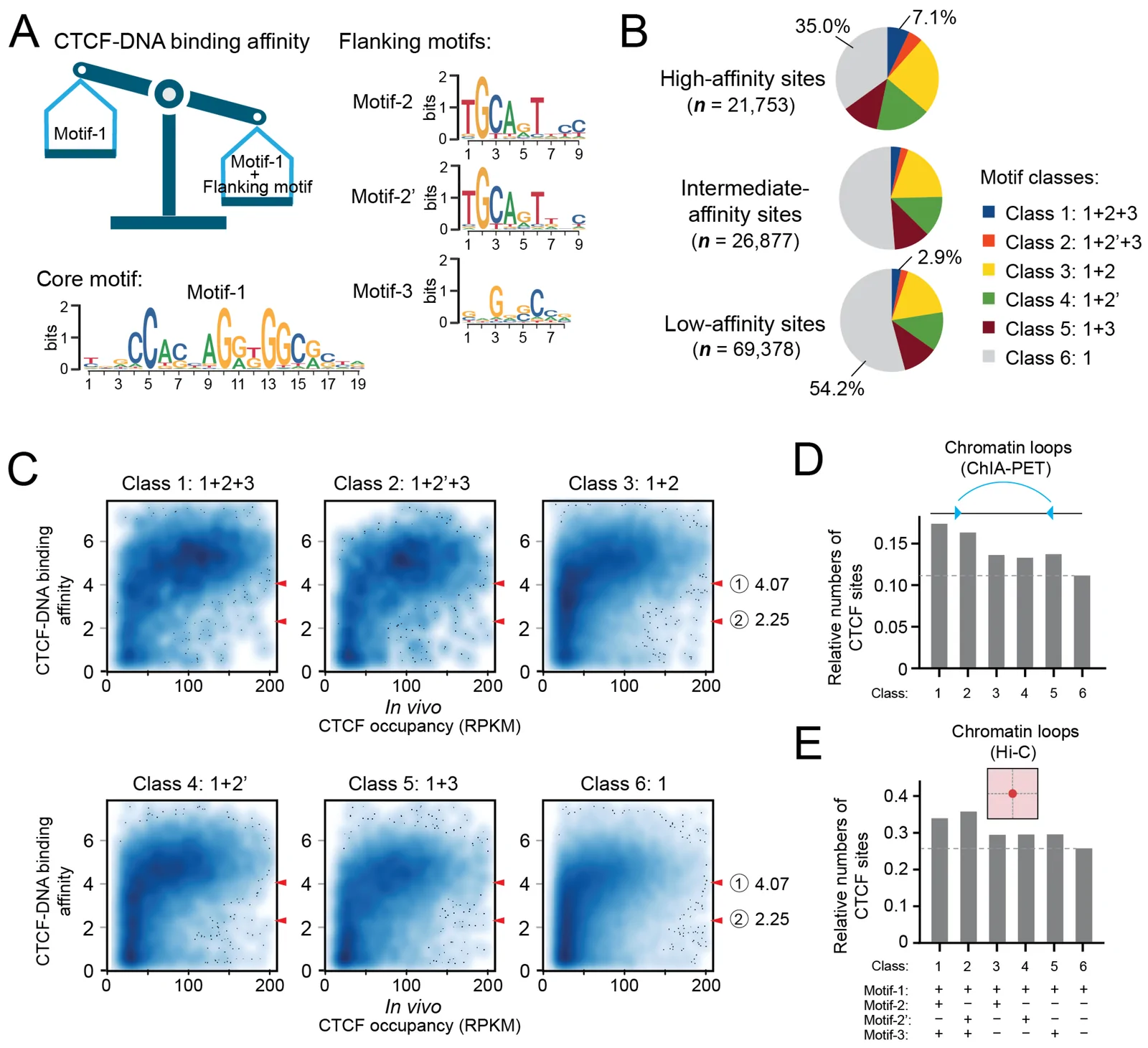
Flanking CTCF motifs are associated with robust CTCF occupancy and strong chromatin loops. (**A**) Schematic illustrating that CTCF sites containing one flanking motif can exhibit higher overall CTCF-DNA binding affinity. The CTCF core motif (Motif-1) and flanking motifs (Motifs-2, -2′, and -3) are shown separately. (**B**) CTCF recognition sequences were classified into three affinity groups (high-, intermediate-, and low-affinity sites) according to the affinity thresholds. The proportions of the six motif-combination classes within each affinity group are shown. (**C**) Smoothed scatter plots showing the relationship between CTCF occupancy in K562 cells and in vitro DNA–CTCF binding affinity for each motif-combination class. (**D**,**E**) Bar plots showing the relative numbers of the six CTCF site classes at CTCF ChIA-PET (**D**) and Hi-C (**E**) loop anchors.

Our recent study showed that removing sequences corresponding to individual CTCF flanking motifs (Motifs-2, -2′, and -3) can significantly reduce CTCF-DNA binding affinity, whereas adding flanking motifs can increase the overall binding affinity of CTCF sites [28] (Figure 7A). To determine whether CTCF motif architecture differed across affinity groups, we first classified CTCF recognition sequences into six motif-architecture classes according to the presence of Motif-1 and its flanking motifs: 1 + 2 + 3, 1 + 2′ + 3, 1 + 2, 1 + 2′, 1 + 3, and Motif-1 only (Figure 7B). We then examined the distribution of these six motif classes across the high-affinity (>4.07), intermediate-affinity (>2.25 and ≤4.07), and low-affinity (≤2.25) groups. Compared with intermediate- and low-affinity sites, high-affinity sites showed greater representation of configurations containing two flanking motifs, with both 1 + 2 + 3 and 1 + 2′ + 3 configurations occurring at more than 1.9-fold higher proportions. Sites containing a single upstream flanking motif (Motif-2 or Motif-2′) were also more prevalent in the high-affinity group, with their proportions being more than 29% higher than those in the lower-affinity groups. In contrast, sites containing only the core Motif-1 were most prevalent among low-affinity sites, accounting for 54.2% of sites compared with only 35.0% in the high-affinity group. Thus, high-affinity sites were more frequently associated with flanking motifs, whereas low-affinity sites were more often composed of the core Motif-1 alone.

To investigate whether flanking-motif architecture was associated with in vivo CTCF occupancy, we examined the relationship between CTCF-DNA binding affinity and CTCF occupancy separately for the six motif classes (Figure 7C). This analysis was restricted to CTCF sites occupied in vivo and excluded unoccupied CTCF recognition sequences. Sites containing two flanking motifs exhibited the highest overall occupancy levels, whereas sites containing only the core Motif-1 exhibited the lowest overall occupancy levels. Together, these results indicate that flanking-motif architecture is associated with more robust CTCF occupancy in vivo.

Given these observations, we hypothesized that CTCF sites containing flanking motifs may preferentially participate in strong chromatin loops. We first analyzed the CTCF ChIA-PET loops used in Figure 6A (Figure 7D). CTCF sites containing two flanking motifs showed the highest frequency of occurrence at strong chromatin loop anchors, followed by sites containing one flanking motif, whose frequency was reduced by an average of 19.6%. Sites containing only the core Motif-1 exhibited the lowest relative frequency, with an average reduction of 33.8% compared with sites containing two flanking motifs. We next applied the same classification scheme to the Hi-C loops analyzed in Figure 6B (Figure 7E). Consistent with the CTCF ChIA-PET results, CTCF sites containing two flanking motifs occurred at the highest frequency at chromatin loop anchors, followed by sites containing one flanking motif, which showed an average reduction of 15.4%. Sites containing only the core Motif-1 again exhibited the lowest frequency, with an average reduction of 26.2% compared with sites containing two flanking motifs. Together, these results indicate that CTCF sites with more extensive flanking motif architectures are preferentially represented at chromatin loop anchors, consistent with an association between flanking CTCF motifs and robust CTCF-associated chromatin interactions.

## 4. Discussion

A central finding of this study is that the relationship between CTCF-DNA binding affinity and in vivo CTCF occupancy is not adequately described by a simple continuum of binding probability. Instead, two quantitative affinity thresholds delineate three affinity-dependent regimes of CTCF occupancy. To assess whether these three regimes were influenced by tissue-specific datasets or redundancy among related cell types, we further removed tissue samples and consolidated redundant cellular datasets, yielding 25 distinct cell types. Although the affinity threshold for the high-affinity group increased modestly from 4.07 to 4.29 after this filtering, high-affinity CTCF sites consistently showed high occupancy frequencies across all 25 cell types, supporting the robustness of the affinity-occupancy relationship across diverse cellular contexts. Notably, the analyzed cell types included tumor-derived and other experimentally derived cellular states, but these diverse cellular contexts provided a broad framework for systematically evaluating CTCF occupancy. We next examined in greater detail the relationship between DNA-encoded binding affinity and in vivo CTCF occupancy within individual cell types. Across cell types, higher DNA-encoded binding affinity was consistently associated with more robust CTCF occupancy. Importantly, even in haploid HAP1 cells, high-affinity sites tended to be occupied at higher frequencies and exhibited stronger overall CTCF occupancy signals. Together, these observations indicate that DNA-encoded binding affinity shapes robust CTCF occupancy across the diverse cellular contexts examined in this study.

Analysis of chromatin compartment transitions provides further support for the affinity-dependent robustness of CTCF occupancy. Switching between A and B compartments was associated with substantial changes in CTCF occupancy at intermediate- and low-affinity sites, whereas high-affinity sites were comparatively insensitive to these transitions. These observations suggest that chromatin context does not influence all CTCF sites equivalently. Instead, the extent to which CTCF occupancy responds to the chromatin environment appears to depend on underlying DNA-binding affinity.

Our synthetic array experiments provide experimental support for these genome-wide observations. These experiments are particularly informative because genomic analyses alone cannot readily distinguish whether high-affinity sites are occupied more frequently because of their DNA sequence or because they preferentially reside in chromatin environments that favor CTCF binding. The synthetic arrays partially decouple these variables by comparing defined affinity classes within a common experimental context. Although lentiviral integration is not completely random and can preferentially target transcriptionally active genomic regions [51], the high-affinity sequence consistently exhibited robust CTCF occupancy across different positions within the tandem array. The persistence of higher occupancy at high-affinity sites therefore supports the conclusion that DNA-encoded affinity contributes to the hierarchy of CTCF occupancy. At the same time, the synthetic array results should not be interpreted as demonstrating that DNA affinity completely determines CTCF occupancy. The lower-affinity sites remained sensitive to cellular context, and the synthetic arrays represent a defined genomic configuration that cannot reproduce the full complexity of endogenous chromatin. Rather, these experiments indicate that affinity provides a reproducible sequence-encoded component of occupancy behavior that operates within, rather than independently of, the broader chromatin environment.

Previous studies have shown that CTCF sites with stronger motif similarity, corresponding to high-affinity CTCF binding, can exhibit more persistent CTCF occupancy and preferentially contribute to higher-order chromatin architecture, whereas sites with weaker motif similarity, corresponding to low-affinity CTCF binding, are enriched at enhancers and promoters and are associated with active chromatin marks and higher transcriptional activity in mouse B cells [32]. Rather than using sensitivity to CTCF depletion or motif similarity as proxies for binding affinity, we used experimentally measured CTCF-DNA binding affinity from MpEMSA-seq [28], and systematically compared it with in vivo CTCF occupancy across diverse human tissue and cellular contexts. Our analysis establishes a quantitative link between experimentally measured CTCF-DNA binding affinity and the robustness of CTCF occupancy, revealing distinct affinity-dependent regimes that distinguish robust from more context-dependent occupancy. Consistent with our findings, previous studies have also reported that a subset of CTCF sites is occupied more frequently across cell types in vivo [52,53], supporting the existence of distinct occupancy behaviors among genomic CTCF sites. Our results further show that these differences in occupancy robustness are quantitatively stratified by DNA-encoded binding affinity, providing a quantitative framework that links CTCF-DNA binding properties encoded in DNA sequence to the stability and context dependence of CTCF occupancy.

We further show that the CTCF-DNA binding affinity may also be associated with the robustness and plasticity of CTCF-mediated 3D chromatin architecture. High-affinity sites were preferentially represented at strong chromatin loop anchors identified by both CTCF ChIA-PET and Hi-C and were more strongly enriched at TAD boundaries, consistent with their more robust occupancy in vivo. Conversely, the greater sensitivity of lower-affinity sites to chromatin compartment transitions suggests that these sites may provide a more context-responsive layer of CTCF occupancy and 3D chromatin interactions. Although our data do not establish that affinity directly determines the stability of individual loops or TAD boundaries, they support a model in which DNA-encoded affinity contributes to the differential sensitivity of CTCF-associated genome organization to chromatin context. Notably, we previously showed that CpG methylation can reduce CTCF-DNA binding strength using in vitro measurements [13]. CpG methylation has also been associated with changes in CTCF occupancy at the genome-wide level [29]. Together, these observations raise the possibility that DNA epigenetic modifications may influence CTCF occupancy and associated chromatin interactions, at least in part, by modulating CTCF-DNA binding affinity. Importantly, such regulation may not require complete inhibition of CTCF binding; instead, a relatively modest reduction in affinity that shifts a site across an affinity threshold could substantially alter its occupancy probability and potentially its associated chromatin interactions. This possibility may also help explain why a subset of high-affinity CTCF sites remains unoccupied in vivo, as chromatin-associated mechanisms could reduce their effective affinity below the range associated with robust occupancy.

This framework may also have implications for interpreting disease-associated genetic variation, much of which is located in noncoding regions [54,55]. A variant may not need to completely disrupt CTCF-DNA binding to substantially alter occupancy; instead, a reduction in affinity that shifts a site from the high-affinity regime below the relevant threshold could markedly reduce its occupancy and potentially affect associated chromatin interactions. More broadly, our findings suggest that a quantitative spectrum of DNA-encoded CTCF-binding affinities may be further modulated by DNA epigenetic modifications and genetic variation, potentially providing a mechanism through which the genome balances relatively stable architectural features with context-dependent remodeling.

Furthermore, our recent study demonstrated that CTCF core motif flanking sequences can contribute to the overall CTCF-DNA binding affinity [28]. The direct interaction of CTCF with flanking DNA sequences is supported by structural studies [36,37,38], in vitro EMSA measurements [16], in vivo ChIP-exo [33] and ChIP-seq data [34,35,36], while their contribution to CTCF-binding affinity is further supported by in vitro MpEMSA-seq measurements and the DeepCTCF deep learning model, which was developed using this high-throughput dataset [28]. In the present study, we found that CTCF sites containing flanking motif sequences exhibited stronger CTCF occupancy and were preferentially represented at strong chromatin interaction anchors. Interestingly, deep learning-based analyses have also identified associations between sequences flanking the CTCF core motif and chromatin loop formation, providing additional support for a potential link between flanking sequence features and higher-order genome organization [56,57]. These findings further extend the affinity-dependent framework proposed here by suggesting that flanking DNA sequences may contribute to chromatin organization through their effects on CTCF-DNA binding affinity. Within this framework, changes in flanking sequences may not need to disrupt CTCF-DNA binding completely; instead, by increasing or decreasing binding affinity sufficiently to shift a site across a functional affinity threshold, they could substantially alter CTCF occupancy and potentially affect associated chromatin interactions.

Several limitations should also be considered. First, the affinity thresholds identified in this study were derived from the relationship between CTCF-DNA binding affinity and CTCF occupancy across the datasets analyzed and therefore may not represent universal boundaries across all cell types or experimental conditions. Although the precise numerical values of these thresholds may vary depending on the datasets and experimental contexts, our analyses consistently revealed two affinity thresholds that delineate three distinct regimes of CTCF occupancy behavior. Thus, the specific threshold values should be interpreted within the quantitative affinity framework established here, whereas the existence of distinct affinity-dependent occupancy regimes appears to be a more general feature. Second, although our synthetic arrays provide experimental evidence that DNA-encoded affinity contributes directly to the hierarchy of CTCF occupancy, they cannot fully recapitulate endogenous genomic environments, including DNA methylation, nucleosome positioning, and neighboring regulatory elements. Third, the association between high-affinity sites and robust CTCF occupancy and higher-order chromatin architecture reflects genome-wide trends and does not by itself establish that affinity is sufficient to determine the stability of individual chromatin loops or TAD boundaries.

Future studies combining quantitative measurements of CTCF-DNA binding affinity with systematic perturbation of DNA methylation, disease-associated genetic variation, and CTCF flanking motifs will be important for determining how changes in affinity quantitatively influence in vivo CTCF occupancy and the formation and persistence of individual chromatin contacts. Direct affinity-altering perturbations at endogenous CTCF sites across different compartment states, coupled with measurements of CTCF occupancy and chromatin contacts, will be important for testing whether changes in CTCF affinity causally influence CTCF occupancy and loop stability. Our previous analyses showed that transcriptionally active subcompartments, particularly A1, were associated with stronger intradomain chromatin interactions and higher NIPBL occupancy [49], while these transcriptionally active regions were also enriched for factors involved in cohesin regulation [58,59,60]. These observations raise the possibility that transcriptionally active chromatin may contribute to stronger chromatin interactions through complementary mechanisms, including enhanced cohesin loading and more stable CTCF occupancy. Understanding how lower-affinity CTCF sites maintain or acquire occupancy within transcriptionally active chromatin, and how such CTCF occupancy contributes to gene expression and cohesin-dependent chromatin interactions, will therefore be an important direction for future studies. Systematically integrating CTCF occupancy with epigenetic features, including histone modifications, chromatin accessibility, DNA methylation, and transcriptional activity, across diverse cellular contexts may further clarify how the local chromatin environment modulates affinity-dependent CTCF occupancy. Such approaches may ultimately enable the predictive design of CTCF-binding elements with defined occupancy properties and more predictable effects on gene regulation and 3D chromatin interactions.

## 5. Conclusions

In summary, our findings establish a quantitative relationship between CTCF-DNA binding affinity and in vivo CTCF occupancy across diverse cellular contexts. Beyond influencing the probability of CTCF occupancy, DNA-encoded binding affinity separates CTCF sites into distinct regimes with different sensitivities to chromatin context. High-affinity sites are more consistently occupied across cellular environments, are less sensitive to chromatin variation, and are preferentially associated with strong chromatin loops and TAD boundaries. In contrast, intermediate- and low-affinity sites exhibit greater context dependence and may provide a more flexible layer of CTCF occupancy and chromatin interactions. We further show that CTCF flanking motifs, which contribute to overall CTCF-DNA binding affinity, are preferentially represented at strong chromatin loop anchors. Together, these findings establish DNA-encoded binding affinity as a quantitative framework for understanding the robustness and context dependence of in vivo CTCF occupancy and suggest that quantitative variation in CTCF-DNA binding affinity may contribute to the balance between robust and context-responsive features of CTCF occupancy and 3D genome organization.

## Data Availability

CTCF-DNA binding affinity data used in this study were downloaded from the open repository Zenodo (https://doi.org/10.5281/zenodo.21410880) [28]. ChIP-seq datasets from 61 ENCODE tissue and cellular contexts were obtained from the ENCODE project [29]. A/B compartment annotations were inferred using the eigenvector function in Juicer from in situ Hi-C datasets [14,45]. Subcompartment annotations were obtained from previously published datasets [46]. CTCF ChIA-PET datasets were downloaded from the UCSC database: https://hgdownload.soe.ucsc.edu/goldenPath/hg19/database/ (accessed on 21 February 2025) [17,27]. The annotation for TAD boundaries was obtained from previously published datasets [47]. The code and processed datasets have been provided at https://github.com/Yin-Zihang/CTCF_affinity (accessed on 7 October 2026).

## Supplementary Materials

The following supporting information can be downloaded at: https://www.mdpi.com/article/10.3390/biology15201843/s1, Figure S1: Sensitivity analyses to evaluate the robustness of the affinity thresholds; Figure S2: Identification of two affinity thresholds that define three CTCF occupancy regimes across 25 non-redundant cell types; Figure S3: Overall distribution of CTCF occupancy for sites with different binding affinities; Figure S4: Distribution of CTCF-occupied sites across subcompartments; Figure S5: Sequences of the synthetic arrays and the three CTCF sites tested experimentally; Table S1: ENCODE CTCF ChIP-seq datasets from 61 tissue and cellular contexts; Table S2: ENCODE CTCF ChIP-seq datasets from 25 non-redundant cell types; Table S3: Primers used in this study; Table S4: Relative CTCF occupancy measured by ChIP-qPCR across two biological replicates.

## Abbreviations

The following abbreviations are used in this manuscript:

DNA	Deoxyribonucleic acid
CTCF	CCCTC-binding factor
TADs	Topologically associating domains
3D	Three-dimensional
ChIP-seq	Chromatin immunoprecipitation sequencing
ChIP-exo	Chromatin immunoprecipitation with exonuclease digestion
ChIP-qPCR	Chromatin immunoprecipitation with quantitative polymerase chain reaction
H3K27ac	Acetylation of lysine 27 on the N-terminal tail of histone H3 protein
H3K4me3	Tri-methylation at the 4th lysine residue on the N-terminal tail of histone H3 protein
H3K4me1	Mono-methylation at the 4th lysine residue on the N-terminal tail of histone H3 protein
ChIA-PET	Chromatin interaction analysis by paired-end tag sequencing
kb	Kilobase
CpG	Cytosine nucleotide followed by a guanine nucleotide
EMSA	Electrophoretic mobility shift assay
MpEMSA-seq	Multiple parallel electrophoretic mobility shift assay followed by high-throughput sequencing
