# Supplementary material for: DNA-Encoded Binding Affinity Shapes Robust CTCF Occupancy Across Cellular Contexts
Source: Biology (Basel). 2026 Oct 14;15(20):1843. doi: 10.3390/biology15201843 (PMC13701538; doi:10.3390/biology15201843)
Supplement: Supplementary file 1 [file biology-15-01843-s001.zip › biology-4595940-supplementary.pdf]

Figures S1-S5, and Tables S1-S4:

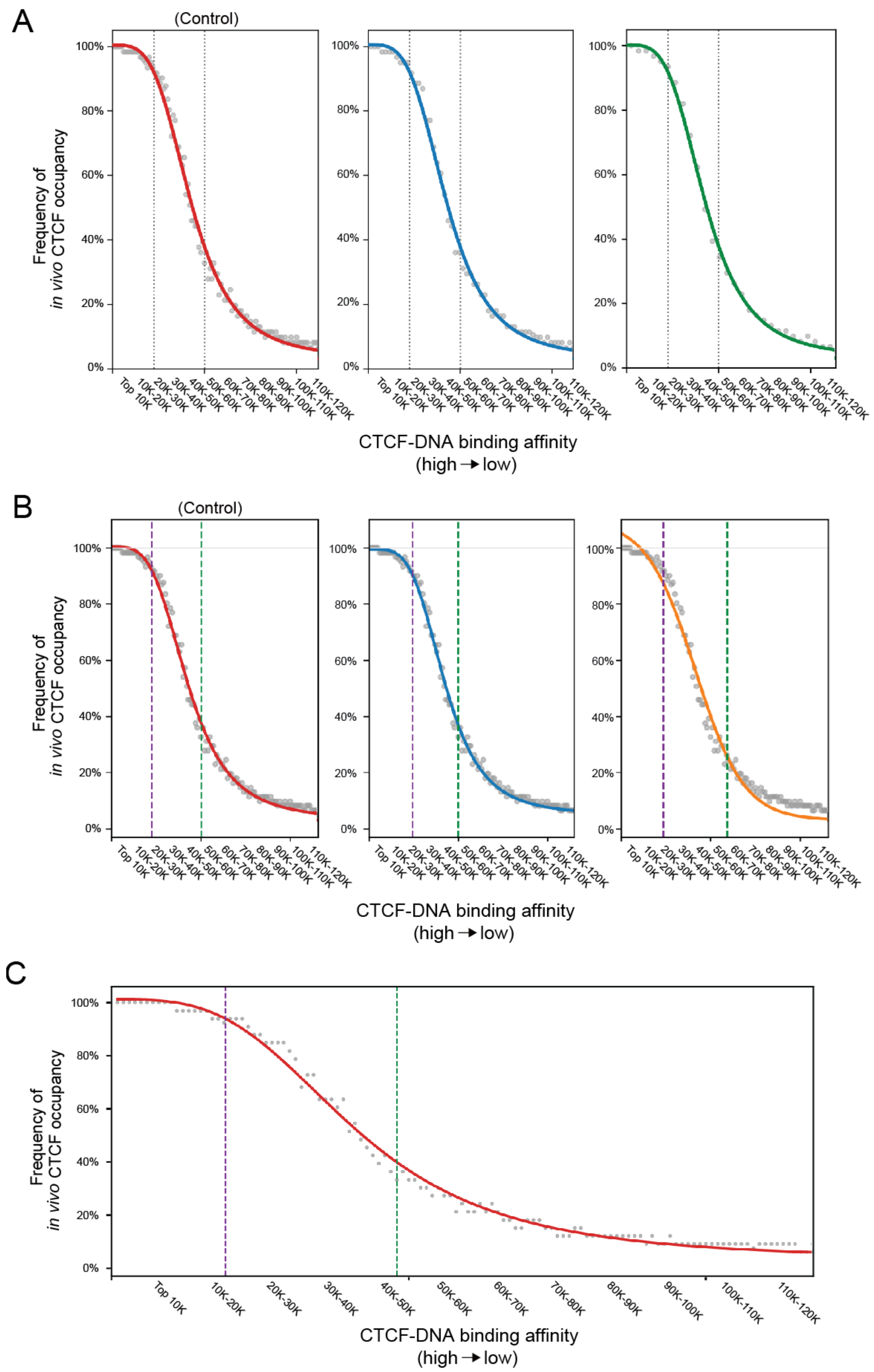

Figure S1. **Sensitivity analyses to evaluate the robustness of the affinity thresholds.** (**A**) Nonlinear regression analysis of the relationship between affinity and occupancy frequency. The fitted regression curves are shown. The left panel shows the fitted curve using the default 120-subset setting, whereas the middle and right panels show the fitted results using 60- and 30-subset analyses, respectively. (**B**) Nonlinear regression analysis using the default fixed-floor model (left), a free-floor model (middle), and a

fixed-floor model in which sites were ranked using their original affinity values rather than log-transformed values (right). (**C**) Only the 33 tissue and cellular contexts in which 60,000-80,000 CTCF sites were identified were included in the downstream analysis. Nonlinear regression analysis of the relationship between affinity and occupancy frequency was performed using the default fixed-floor model. The two inflection points, corresponding to the affinity thresholds, are indicated by dashed lines.

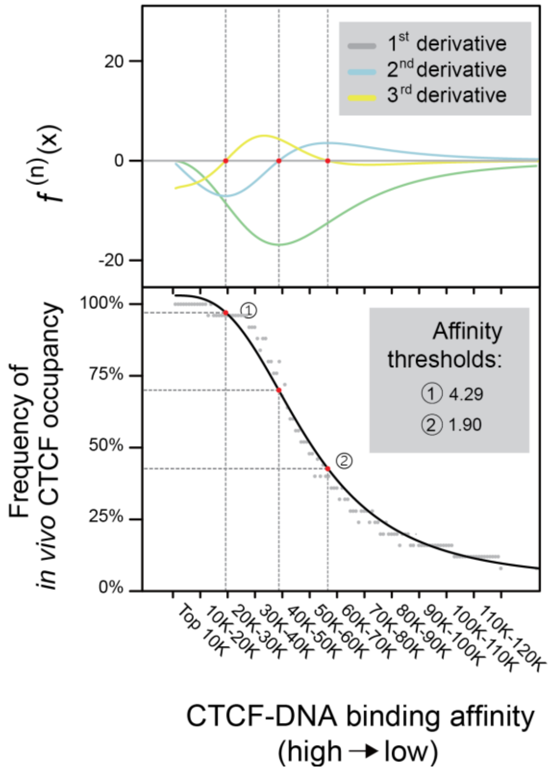

Figure S2. **Identification of two affinity thresholds that define three CTCF occupancy regimes across 25 non-redundant cell types.** Nonlinear regression analysis of the relationship between affinity and occupancy frequency (bottom) and the corresponding first-, second-, and third-order derivative curves (top). The fitted regression curve is shown in black and the original data points in grey. The two inflection points, corresponding to affinity thresholds of 4.29 and 1.90, are indicated.

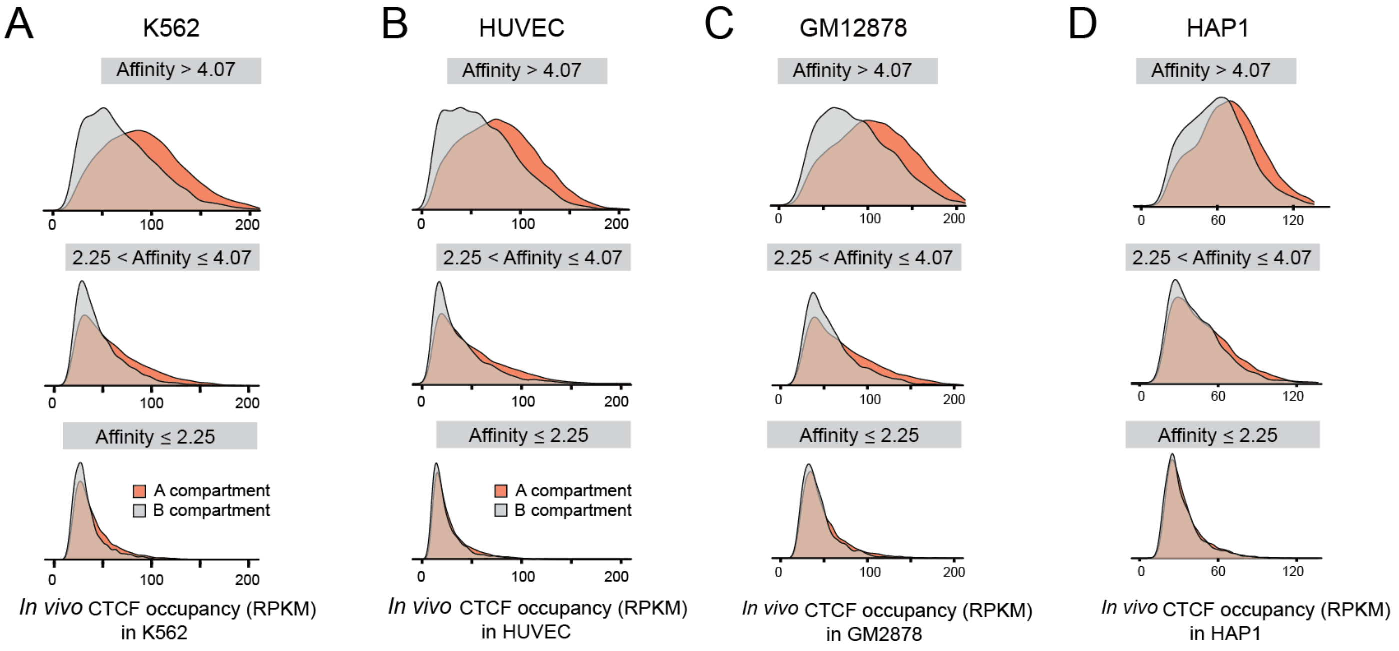

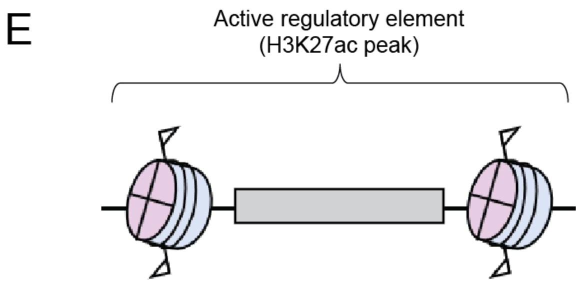

F

Affinity of CTCF sites:	High	Intermediate	Low
K562	3.87% (772)	5.62% (980)	13.44% (2,324)
GM12878	4.76% (854)	7.99% (981)	21.63% (1,628)

Figure S3. Overall distribution of CTCF occupancy for sites with different binding affinities. (**A-D**) Density plots show the overall distributions of high-affinity (top), intermediate-affinity (middle), and low-affinity (bottom) CTCF sites located in the A (orange) or B (gray) compartments across CTCF occupancy levels. (**E**) Schematic illustration of an active regulatory element defined by an H3K27ac peak. (**F**) Proportion of high-, intermediate-, and low-affinity CTCF sites located within active regulatory elements in K562 and GM12878 cells. The numbers in parentheses indicate the number of CTCF sites.

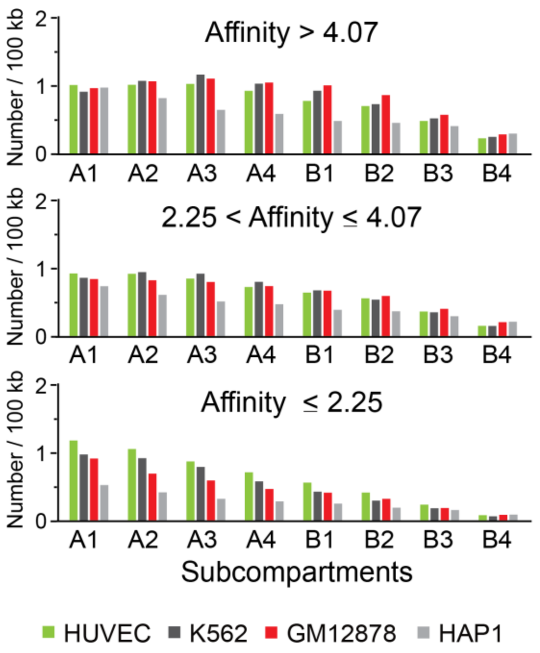

Figure S4. Distribution of CTCF-occupied sites across subcompartments. Bar plots show the average number of CTCF-occupied sites per 100-kb genomic region assigned to the A1, A2, A3, A4, B1, B2, B3, or B4 subcompartments in HUVEC (green), K562 (dark gray), GM12878 (red), and HAP1 (light gray) cells.

A

```
ATCGATacatactttcctgaagaggtcgtcgaacacatttttagcttcctgcctgcacag
agagatagaaacacagtgagcctggtctgcaaagtgtggtacgagatcgaacgcctgagc
cggagaggagtgttcgtcggcaactgctatgctgtgagagcaggcagggtcgccgctagg
tttccaaatgtgcgcgcactgaccgtcaaggggaaaccccacttcgccgactttaacctg
gtgcaccatgattggggaggatacgccggcccttggatcgaggcagccgctcgcggctgt
catggactGAATTC(#1)agcgaatggtggtctctgacgaaAgtctggagctgctggctc
ggagcttccctaggtttcgcgcactggtgctgatttcttgcgaaggcttcagcaccgatg
gactggcagccgtggcctcccactgtaagctgctgcgggagctggacctccaggagaatg
aagtggaggatagaggacccagatagctgtcttgcttcccagactcatgtaccagcctgg
tgtccctgaactttgcctgcatcaaaggcgaagtgaatgctgggtccctggagcggctgg
tctcaagaagccccaacctgaggtctctgcggctgaaccggagcgtgagcgtggacactc
tggctaagattctgctgagaacccctaacctggaggatctgggaaccggcaatctgacag
acgatttccagacagaatcctactttaaactgacttctgccctggagaagtgtaaaatgc
tgaggagtctgtcaggattctgggatgcttcacccgtgtgcctgagctttatctaccctc
tgtgtgcacagctgacaggcctgaacctgagctatgcaccaaccctggacgccagtgtaa
tgacaaagatgatctcacgcTgcgtgaaactccagcgactgtgggtgctggactgt(#2)
aggacatccaggaactgagagtgttcccatctgatctatacgtggccggatatagaatcg
tcactgaggaaggcctggtggcagtctcactgggatgcccaaagctgaacagcctgctgt
atttctgtcatcagatgactaatgctgcactggtgaccgtcgccaagaactgccctaatt
tcacccgatttcggctgtgtattctggaaccaggcaaacccgacgtggtcacatcccagc
cactggatgaagggtttggagctatcgtgagagagtgcaagggactccagaggctgagca
tttccggcctgctgacagacaaagtgttcatgtacatcggcaagtatgctaagcagctgg
agatgctgagcattgcatttgccggagactccgataagggcatgatgcacgtgatgaacg
ggtgtaagaatctgcgaaaactggaaatccgggacagccctttcggggatgccgctctgc
tgggaaactttgccagatacgagacaatgaggagcctgtggatgtctagttgcaatgtga
ctctgaagggctgtcaggtcctggctagtaaaatgcctatgctgaacgtggaagtcatta
atgagcgggacgggtctaacgaaatggaggaaaatcatggcgacctgccaaaggtggaga
aactgtatgtgtatcggaccaccg(#3)CTCGAGtgctcccaactttgtgaagattctgt
gaggatccatcggatcccgggcacgtcgacagtaccgcgggcccgggatccaccggggcc
gcgactctagatcataatcagccataccacatttgtagaggttttacttgctttaaaaaa
cctcccacacctccccctgaacctgaaacataaaatgaatgcaattgttgttGTTAAC
```

B

High-affinity CTCF site: TGCAATTCCAAATCTTGCCACCAGGTGGTGGTGGCATGTTA

Intermediate-affinity CTCF site: TGCCTGGCCACTCTTGGCCACAGGGTGTCATCATCCCACAA

Low-affinity CTCF site: AGAAAAGAATGAAGAAGCCACATGGGGGCATAACTGCCTCA

Figure S5. Sequences of the synthetic arrays and the three CTCF sites tested experimentally. (**A**) Sequences of the synthetic arrays, with the three positions at which CTCF sites were inserted indicated as (#1), (#2), and (#3). The restriction enzyme recognition sites, used in the molecular cloning constructs, are indicated in uppercase letters. (**B**) Sequences of the three CTCF sites tested experimentally.

Table S1. ENCODE CTCF ChIP-seq datasets from 61 tissue and cellular contexts.

ID	Term	Category	Original downloaded file	Descriptions of culture conditions
1	Wi-38	fibroblast	UwTfbsWi38CtcfStdPk	Adherent WI-38/hTERT/GFP-RAF-ER cells. MEM (Earle salts, 2 mM L-glutamine), 10% FBS, 1× NEAA, 1 mM sodium pyruvate and 1× P/S; 37°C, 5% CO_2.
2	Weri-rb-1	retinoblastoma	UwTfbsWerirb1CtcfStdPk	Suspension culture in RPMI-1640 (2 mM L-glutamine), 10% FBS and 1× P/S; 37°C, 5% CO_2.
3	Spleen_oc	spleen tissue	OpenChromChipSpleenocCtcfPk	Not applicable: primary frozen tissue; no in vitro culture.
4	Saec	epithelial	UwTfbsSaecCtcfStdPk	Adherent culture in Lonza SAGM BulletKit CC-4175 (CC-3119 + CC-4124); 37°C, 5% CO_2.
5	Rptec	epithelial	UwTfbsRptecCtcfStdPk	Adherent culture in Lonza REGM BulletKit CC-3190 (CC-3191 + CC-4127); 37°C, 5% CO_2.
6	Progfib	fibroblast	OpenChromChipProgfibCtcfPk	Adherent HGADFN167/HGPS fibroblasts. MEM (Invitrogen 10370-021), 15% FBS and 1% P/S; 37°C, 5% CO_2.
7	Pancreas_oc	pancreas tissue	OpenChromChipPancreasocCtcfPk	Not applicable: primary frozen tissue; no in vitro culture.
8	Nhlf	fibroblast	UwTfbsNhlfCtcfStdPk	Adherent culture in Lonza FGM-2 BulletKit CC-3132 (FBM CC-3131 + CC-4126); 37°C, 5% CO_2.
9	Nhek	skin epidermal keratinocytes	UwTfbsNhekCtcfStdPk	Adherent culture in Lonza KGM BulletKit CC-3111 (CC-3101 + CC-4131); 37°C, 5% CO_2.
10	Nhdf-neo	fibroblast	UwTfbsNhdfneoCtcfStdPk	Adherent culture in Lonza FGM-2 BulletKit CC-3132 (CC-3131 + CC-4126); 37°C, 5% CO_2.
11	Nb4	leukemia	UwTfbsNb4CtcfStdPk	Suspension culture in RPMI-1640 (2 mM L-glutamine), 10% FBS and 1× P/S; 37°C, 5% CO_2.

ID	Term	Category	Original downloaded file	Descriptions of culture conditions
12	Medullo	medulloblastoma	OpenChromChipMedulloCtcfPk	D721 medulloblastoma cell line, grown as suspension aggregates, not direct tumor tissue. 1× Improved MEM Zinc Option (86-0194DJ), supplemented per liter with 10 mL HEPES and 40 mL 5.5% $NaHCO_3$, plus 10% FBS; 37°C, 5% CO_2.
13	Mcf-7	adenocarcinoma	UwTfbsMcf7CtcfStdPk	Adherent culture in MEM (2 mM L-glutamine), NEAA, 10% FBS, 1× P/S and 1.5 g/L $NaHCO_3$; 37°C, 5% CO_2.
14	Lung_oc	lung tissue	OpenChromChipLungocCtcfPk	Not applicable: primary frozen tissue; no in vitro culture.
15	Lncap	adenocarcinoma	OpenChromChipLncapCtcfPk	Adherent culture in RPMI-1640, 10% FBS and Plasmocin (0.5 mg/500 mL); 37°C, 5% CO_2.
16	Kidney_oc	kidney tissue	OpenChromChipKidneyocCtcfPk	Not applicable: primary frozen tissue; no in vitro culture.
17	K562	leukemia	UwTfbsK562CtcfStdPk	Suspension culture in RPMI-1640 and 10% FBS, 2 mM L-glutamine, 1× P/S and 10 mM sodium pyruvate; 37°C, 5% CO_2.
18	Imr90-bIggrab	fibroblast	SydhTfbsImr90CtcfbIggrabPk	Adherent culture in MEM (Earle salts, 2 mM L-glutamine), 10% heat-inactivated FBS, 1× NEAA, 1 mM sodium pyruvate and 1× P/S; 37°C, 5% CO_2.
19	Hvmf	fibroblast	UwTfbsHvmfCtcfStdPk	Adherent culture in ScienCell FM 2301 on poly-L-lysine (2 μg/cm^2); 37°C, 5% CO_2.
20	Huvec	epithelial	UwTfbsHuvecCtcfStdPk	Adherent culture in Lonza EGM-2 BulletKit CC-3162 (CC-3156 + CC-4176); 37°C, 5% CO_2.
21	HrpepiC	epithelial	UwTfbsHrpeCtcfStdPk	Adherent culture in ScienCell EpiCM 4101 on poly-L-lysine (2 μg/cm^2); 37°C, 5% CO_2.
22	Hre	epithelial	UwTfbsHreCtcfStdPk	Adherent culture in Lonza REGM BulletKit CC-3190 (CC-3191 + CC-4127); 37°C, 5% CO_2.

ID	Term	Category	Original downloaded file	Descriptions of culture conditions
23	Hpf	fibroblast	UwTfbsHpfCtcfStdPk	Adherent culture in ScienCell FM 2301 on poly-L-lysine (2 μg/cm^2); 37°C, 5% CO_2.
24	Hpaf	fibroblast	UwTfbsHpafCtcfStdPk	Adherent culture in ScienCell FM 2301 on poly-L-lysine (2 μg/cm^2); 37°C, 5% CO_2.
25	Hmf	fibroblast	UwTfbsHmfCtcfStdPk	Adherent culture in ScienCell FM 2301 on poly-L-lysine (2 μg/cm^2); 37°C, 5% CO_2.
26	Hmec	epithelial	UwTfbsHmecCtcfStdPk	Adherent culture in Lonza MEGM BulletKit CC-3150 (CC-3151 + CC-4136); 37°C, 5% CO_2.
27	Hl-60	leukemia	UwTfbsHl60CtcfStdPk	Suspension culture in DMEM (2 mM L-glutamine), 20% FBS and 1× P/S; 37°C, 5% CO_2.
28	Hff	fibroblast	UwTfbsHffCtcfStdPk	Adherent culture in DMEM (4.5 g/L glucose, L-glutamine and sodium pyruvate), 10% FBS and 1× P/S; 37°C, 5% CO_2.
29	Hepg2	hepatocellular carcinoma	UwTfbsHepg2CtcfStdPk	Adherent culture in MEM (2 mM L-glutamine), NEAA, 1 mM sodium pyruvate, 10% FBS, 1× P/S and 1.5 g/L $NaHCO_3$, at 37°C, 5% CO_2.
30	Hela-s3	cervical carcinoma	UwTfbsHelas3CtcfStdPk	Adherent culture in DMEM (2 mM L-glutamine), 10% FBS and 1× P/S, at 37°C, 5% CO_2.
31	Hek293	kidney tissue	UwTfbsHek293CtcfStdPk	Adherent culture in MEM (2 mM L-glutamine), NEAA, 1 mM sodium pyruvate, 10% FBS, 1× P/S and 1.5 g/L $NaHCO_3$; 37°C, 5% CO_2.
32	HeepiC	epithelial	UwTfbsHeeCtcfStdPk	Adherent culture in ScienCell EpiCM2 4121 on poly-L-lysine (2 μg/cm^2); 37°C, 5% CO_2.
33	Hct-116	colorectal carcinoma	UwTfbsHct116CtcfStdPk	Adherent culture in modified McCoy's 5A medium, 10% FBS and 1× P/S; 37°C, 5% CO_2.
34	HcpepiC	epithelial	UwTfbsHcpeCtcfStdPk	Adherent culture in ScienCell EpiCM 4101 on poly-L-lysine (2 μg/cm^2); 37°C, 5% CO_2.

ID	Term	Category	Original downloaded file	Descriptions of culture conditions
35	Hcm	cardiac myocytes	UwTfbsHcmCtcfStdPk	Adherent culture in ScienCell CMM 6201 on poly-L-lysine (2 μg/cm²); 37°C, 5% CO_2.
36	Hcfaa	fibroblast	UwTfbsHcfaaCtcfStdPk	Adherent culture in ScienCell FM 2301 on poly-L-lysine (2 μg/cm²); 37°C, 5% CO_2.
37	Hbmec	brain microvascular endothelial cells	UwTfbsHbmecCtcfStdPk	Adherent culture in ScienCell ECM 1001 on fibronectin (2 μg/cm²); 37°C, 5% CO_2. Seed at 7,500 cells/cm²; passage at 80% confluence.
38	Ha-sp	astrocytes	UwTfbsHaspCtcfStdPk	Adherent culture in ScienCell AM 1801 on poly-L-lysine (2 μg/cm²); 37°C, 5% CO_2.
39	Hac	astrocytes	UwTfbsHacCtcfStdPk	Adherent culture in ScienCell AM 1801 on poly-L-lysine (2 μg/cm²); 37°C, 5% CO_2.
40	H1hesc	embryonic stem cells	OpenChromChipH1hescCtcfPk	Batch-specific culture conditions are not provided in ENCODE/GEO.
41	Gm20000	lymphoblastoid	OpenChromChipGm20000CtcfPk	EBV-transformed B-lymphoblastoid cells in suspension. RPMI-1640, 2 mM L-glutamine, 15% FBS and 1× P/S; 37°C, 5% CO_2.
42	Gm19240	lymphoblastoid	OpenChromChipGm19240CtcfPk	EBV-transformed B-lymphoblastoid cells in suspension. RPMI-1640, 2 mM L-glutamine, 15% FBS and 1× P/S; 37°C, 5% CO_2.
43	Gm13977	lymphoblastoid	OpenChromChipGm13977CtcfPk	EBV-transformed B-lymphoblastoid cells in suspension. RPMI-1640, 2 mM L-glutamine, 15% FBS and 1× P/S; 37°C, 5% CO_2.
44	Gm13976	lymphoblastoid	OpenChromChipGm13976CtcfPk	EBV-transformed B-lymphoblastoid cells in suspension. RPMI-1640, 2 mM L-glutamine, 15% FBS and 1× P/S; 37°C, 5% CO_2.
45	Gm12892	lymphoblastoid	OpenChromChipGm12892CtcfPk	EBV-transformed B-lymphoblastoid cells in suspension. RPMI-1640, 2 mM L-glutamine, 15% FBS and 1× P/S; 37°C, 5% CO_2.

ID	Term	Category	Original downloaded file	Descriptions of culture conditions
46	Gm12878	lymphoblastoid	UwTfbsGm12878CtcfStdPk	EBV-transformed B-lymphoblastoid cells in suspension. RPMI-1640, 2 mM L-glutamine, 15% FBS and 1× P/S; 37°C, 5% CO_2.
47	Gm12801	lymphoblastoid	UwTfbsGm12801CtcfStdPk	EBV-transformed B-lymphoblastoid cells in suspension. RPMI-1640, 2 mM L-glutamine, 15% FBS and 1× P/S; 37°C, 5% CO_2.
48	Gm10248	lymphoblastoid	OpenChromChipGm10248CtcfPk	EBV-transformed B-lymphoblastoid cells in suspension. RPMI-1640, 2 mM L-glutamine, 15% FBS and 1× P/S; 37°C, 5% CO_2.
49	Gm06990	lymphoblastoid	UwTfbsGm06990CtcfStdPk	EBV-transformed B-lymphoblastoid cells in suspension. RPMI-1640, 2 mM L-glutamine, 15% FBS and 1× P/S; 37°C, 5% CO_2.
50	Gliobla	glioblastoma	OpenChromChipGlioblaCtcfPk	Adherent D54/H54 glioblastoma cells. 1× Improved MEM Zinc Option (86-0194DJ), supplemented per liter with 10 mL HEPES and 40 mL 5.5% $NaHCO_3$, plus 10% FBS and 2% P/S; 37°C, 5% CO_2.
51	Fibrobl	fibroblast	OpenChromChipFibroblCtcfPk	Adherent AG08470 normal child fibroblasts (female donor). MEM (Invitrogen 10370-021), 15% FBS and 1% P/S; 37°C, 5% CO_2.
52	Caco-2	adenocarcinoma	UwTfbsCaco2CtcfStdPk	Adherent culture in MEM (2 mM L-glutamine), NEAA, 1 mM sodium pyruvate, 20% FBS, 1× P/S and 1.5 g/L $NaHCO_3$; 37°C, 5% CO_2.
53	Bj	fibroblast	UwTfbsBjCtcfStdPk	Adherent BJ cells (ATCC CRL-2522). MEM (2 mM L-glutamine), 10% FBS, 1 mM sodium pyruvate, NEAA, 1× P/S and 1.5 g/L $NaHCO_3$; 37°C, 5% CO_2.
54	BE2_C	neuroblastoma	UwTfbsBe2cCtcfStdPk	Adherent culture in F-12K/MEM (1:1; MEM contains Earle salts and 2 mM L-glutamine), 10% FBS, 1 mM sodium pyruvate, 1× NEAA and 1× P/S; 37°C, 5% CO_2.
55	Aoaf	fibroblast	UwTfbsAoafCtcfStdPk	Adherent culture in Lonza SCGM BulletKit CC-3205 (CC-3204 + CC-4181); 37°C, 5% CO_2.

ID	Term	Category	Original downloaded file	Descriptions of culture conditions
56	Ag10803	fibroblast	UwTfbsAg10803CtcfStdPk	Adherent fibroblasts. Eagle MEM (Earle salts and L-glutamine), 15% FBS, 1× NEAA and 1× P/S; 37°C, 5% CO_2.
57	Ag09319	fibroblast	UwTfbsAg09319CtcfStdPk	Adherent fibroblasts. Eagle MEM (Earle salts and L-glutamine), 15% FBS, 1× NEAA and 1× P/S; 37°C, 5% CO_2.
58	Ag09309	fibroblast	UwTfbsAg09309CtcfStdPk	Adherent fibroblasts. Eagle MEM (Earle salts and L-glutamine), 15% FBS, 1× NEAA and 1× P/S; 37°C, 5% CO_2.
59	Ag04450	fibroblast	UwTfbsAg04450CtcfStdPk	Adherent fibroblasts. Eagle MEM (Earle salts and L-glutamine), 15% FBS, 1× NEAA and 1× P/S; 37°C, 5% CO_2.
60	Ag04449	fibroblast	UwTfbsAg04449CtcfStdPk	Adherent fibroblasts. Eagle MEM (Earle salts and L-glutamine), 15% FBS, 1× NEAA and 1× P/S; 37°C, 5% CO_2.
61	A549	epithelial	UwTfbsA549CtcfStdPk	Adherent culture in F-12K (ATCC 30-2004), 10% FBS and 1× P/S; 37°C, 5% CO_2.

Table S2. ENCODE CTCF ChIP-seq datasets from 25 non-redundant cell types.

ID	Term	Original Downloaded File	Description
1	Wi-38	UwTfbsWi38CtcfStdPk	Fibroblast
2	Weri-rb-1	UwTfbsWerirb1CtcfStdPk	Retinoblastoma cell
3	Saec	UwTfbsSaecCtcfStdPk	Small airway epithelial cell
4	Rptec	UwTfbsRptecCtcfStdPk	Renal epithelial cell
5	Nhek	UwTfbsNhekCtcfStdPk	Keratinocyte
6	Nb4	UwTfbsNb4CtcfStdPk	Promyelocytic leukemia cell
7	Mcf-7	UwTfbsMcf7CtcfStdPk	Breast adenocarcinoma cell
8	Lncap	OpenChromChipLncapCtcfPk	Prostate adenocarcinoma cell
9	K562	UwTfbsK562CtcfStdPk	K562 myeloid/erythroid leukemia cell
10	Huvec	UwTfbsHuvecCtcfStdPk	Endothelial cell
11	HrpepiC	UwTfbsHrpeCtcfStdPk	Retinal pigment epithelial cell
12	Hmec	UwTfbsHmecCtcfStdPk	Mammary epithelial cell
13	Hepg2	UwTfbsHepg2CtcfStdPk	Hepatocellular carcinoma cell
14	Hela-s3	UwTfbsHelas3CtcfStdPk	Cervical carcinoma cell
15	Hek293	UwTfbsHek293CtcfStdPk	HEK293 transformed cell
16	HeepiC	UwTfbsHeeCtcfStdPk	Esophageal epithelial cell
17	Hct-116	UwTfbsHct116CtcfStdPk	Colorectal carcinoma cell
18	HcpepiC	UwTfbsHcpeCtcfStdPk	Choroid plexus epithelial cell
19	Hcm	UwTfbsHcmCtcfStdPk	Cardiomyocyte
20	Hac	UwTfbsHacCtcfStdPk	Astrocyte
21	H1hesc	OpenChromChipH1hescCtcfPk	Embryonic stem cell
22	Gm12878	UwTfbsGm12878CtcfStdPk	B lymphoblastoid cell

23	Gliobla	OpenChromChipGlioblaCtcfPk	Glioblastoma cell
24	BE2_C	UwTfbsBe2cCtcfStdPk	Neuroblastoma cell
25	A549	UwTfbsA549CtcfStdPk	Lung adenocarcinoma cell

Table S3. Primers used in this study.

Primer Name	5'-3' sequence
**Primers used for molecular cloning:**	
SMW-F1	GGAATTCTGCAATTCCAAATCTTGCCACCAGGTGGTGGTGGCATGTTAAGCGAATGGTGGTCTCTGAC
SMW-R1	TGGGATGATGACACCCTGTGGCCAAGAGTGGCCAGGCAACAGTCCAGCACCCACAGTC
SMW-F2	GCCACAGGGTGTCATCATCCCACAAAGGACATCCAGGAACTGAGAG
SMW-R2	CCTCGAGTGAGGCAGTTATGCCCCCATGTGGCTTCTTCATTCTTTTCTCGGTGGTCCGATACACATAC
WMS-F1	GGAATTCAGAAAAGAATGAAGAAGCCACATGGGGGCATAACTGCCTCAAGCGAATGGTGGTCTCTGAC
WMS-R2	CCTCGAGTAACATGCCACCACCACCTGGTGGCAAGATTTGGAATTGCACGGTGGTCCGATACACATAC
SWM-R1	CCCATGTGGCTTCTTCATTCTTTTCTACAGTCCAGCACCCACAGTC
SWM-F2	AGAATGAAGAAGCCACATGGGGGCATAACTGCCTCAAGGACATCCAGGAACTGAGAG
SWM-R2	CCTCGAGTTGTGGGATGATGACACCCTGTGGCCAAGAGTGGCCAGGCACGGTGGTCCGATACACATAC
MWS-F1	GGAATTCTGCCTGGCCACTCTTGGCCACAGGGTGTCATCATCCCACAAAGCGAATGGTGGTCTCTGAC
MSW-R1	CACCTGGTGGCAAGATTTGGAATTGCAACAGTCCAGCACCCACAGTC
MSW-F2	TCCAAATCTTGCCACCAGGTGGTGGTGGCATGTTAAGGACATCCAGGAACTGAGAG
**Primers used for ChIP-qPCR:**	
#1F	CACTTCGCCGACTTTAACCTG
#1R	AGCCTTCGCAAGAAATCAGC
#2F	CGCCAGTGTAATGACAAAGATG
#2R	GCCTTCCTCAGTGACGATTC
#3F	GACGGGTCTAACGAAATGGAG
#3R	CAAATGTGGTATGGCTGATTATG
Postive-F	CTTTGGGTAGCCGTCAAGAATAC
Postive-R	AGATGACTGAAGGACAGATGGG
Negative-F	GTTGACCGAATCACCGACCT
Negative-R	ACCCACACCTTGCCGATGTC
Normalized-F	TCCACTTCTCTCCTTTTGCG
Normalized-R	TCATTCACTCATTCAACAGACG

Table S4. Relative CTCF occupancy measured by ChIP-qPCR across two biological replicates.

Groups		#1	#2	#3	Negative control	Positive control
S-S-S	Replicate 1	0.48605	0.69954	0.67537	0.11564	0.73889
	Replicate 2	0.64256	1.09353	1.40337	0.22007	0.96763
S-M-L	Replicate 1	0.49617	0.02578	0.06876	0.01888	0.89918
	Replicate 2	0.70346	0.38008	0.29937	0.14276	1.19865
S-L-M	Replicate 1	0.44171	0.00749	0.07293	0.02642	0.62894
	Replicate 2	0.88219	0.25026	0.32611	0.26280	0.91585
L-S-M	Replicate 1	0.19297	0.58445	0.33933	0.13841	0.97920
	Replicate 2	0.43568	0.57076	0.44022	0.20062	0.93882
M-S-L	Replicate 1	0.16345	0.58931	0.34663	0.17947	0.93102
	Replicate 2	0.38725	0.61691	0.41284	0.22000	0.97938
M-L-S	Replicate 1	0.12019	0.17956	0.83353	0.29950	1.03384
	Replicate 2	0.26089	0.19767	1.07698	0.12041	0.90611
L-M-S	Replicate 1	0.14593	0.16230	0.70345	0.19279	0.97388
	Replicate 2	0.23979	0.16222	0.94664	0.12570	0.89064
